# The Impact of Reassurance on Musculoskeletal (MSK) Pain: A Qualitative Review

**DOI:** 10.3390/bs11110150

**Published:** 2021-10-30

**Authors:** Lok Cheung, Andrew Soundy

**Affiliations:** School of Sport, Exercise and Rehabilitation Sciences, University of Birmingham, Birmingham B15 2TT, UK; LHC808@student.bham.ac.uk

**Keywords:** reassurance, musculoskeletal pain, review, qualitative, patient-therapist interaction

## Abstract

Background: The reassurance provided during patient-therapist interactions is significantly associated with psychosocial outcomes, including fear and increased confidence. Currently, there are no available reviews that discuss the impact of reassurance for patients with musculoskeletal (MSK) pain. The aim of the present review was to qualitatively synthesize themes around reassurance mechanisms, and the impact during the interaction between patients with MSK pain and therapists. A systematic search strategy was undertaken. Studies were included if they were qualitative or mixed methods studies, examining the patient-therapist consultation, in any MSK clinical setting, with any health care professional, for adult patients with acute to chronic MSK pain. A thematic synthesis was conducted and supported by a particular assessment using CERQual. Results: Twenty-four studies were included (451 patients). Certain themes that related to both positive and negative outcomes of reassurance were identified as well as themes that illustrate the mechanisms causative of the outcomes. Using CerQual, we identified the most supported outcomes. Conclusions: Effective reassurance includes affectionate interactions such as therapeutic relationship building and interpersonal skills, an individualized patient-centered approach, education and the provision of self-management strategies. It should be noted that some interactions that use pathoanatomic models led patients to misinterpret the information provided, this created feelings of fear.

## 1. Introduction

Musculoskeletal (MSK) pain conditions represent pain that is generated from affected bone(s), muscle(s), joints(s), or related soft tissue(s). This means that it is limited to nociceptive pain [1], pain is classified according to its duration; acute pain is classified as being present for <3 months [2]. MSK pain is the most common contributor to disability across the globe, with between one in three and one in five people of all ages, living with MSK pain [3]. MSK pain conditions have a major impact on a person’s livelihood, for instance, individuals with MSK pain conditions are more likely to be out of work than individuals without MSK pain conditions, and subsequently, are more likely to retire younger [4]. MSK pain implicates a high financial burden. For instance, MSK pain conditions cost the United Kingdom £100 billion annually [4]. In order to reduce the burden caused by MSK pain on society, research better improves the effectiveness of MSK pain management is necessary [3]. The inability to cure MSK pain means that individuals can be caught in a vicious cycle, which demands frequent health care visits [5]. Health care professionals are required to understand the value of reassurance during the clinical encounter [6]. Reassurance forms the main component of communication required for MSK pain encounters [7]. It is essential that the two major types of reassurance are understood.

Affective Reassurance (AR) includes the following aspects of a clinical interaction: creating rapport, displaying empathy, reducing patient anxiety (relaxation), and making the patient feeling cared for and understood, with verbal and non-verbal behaviour [8,9]. Cognitive Reassurance (CR) includes the following aspects of a clinical interaction: providing explanations and education—improving the patient’s knowledge and understanding of their condition through education, to create a stable systematic change in their belief over time, while improving patient satisfaction and empowerment [9]. The central aspects of AR and CR highlight the importance of reassurance for patient-centered care, and the need for research in reassurance as there is currently very limited evidence available to identify how best to provide AR or CR and why different components are important to clinical practice [10].

Systematic review evidence has identified that CR has been associated with significantly higher improvements in patient satisfaction and enablement (a patient’s perception of being empowered to act to improve their condition following an interaction) [8], and as having a positive effect on treatment outcomes [11]. However, current reviews [8,11] have not been able to consider the quality of evidence or identify how to provide AR and CR to patients with MSK pain. This lacuna has been recognized in recent literature and, to the best of the authors’ knowledge, systematic reviews on the direct implications of CR and AR for the impact on individuals with MSK pain do not yet exist. Qualitative research is well suited to help develop our understanding, due to the multifaceted nature of AR and CR and the ability of qualitative research to identify perceived impacts and how and why (mechanisms) they may occur. The value and need for such a review is supported by review evidence in other conditions e.g., [12]. Given this, the objective of this review is to consider the impact and the mechanisms of reassurance on patient-therapist interactions using qualitative data from musculoskeletal pain settings.

## 2. Materials and Methods

### 2.1. Protocol

This review was conducted in accordance with the PRISMA 2020 statement [13], as well as The Enhancing Transparency in Reporting the Synthesis of Qualitative Research (ENTREQ) statement [14].

### 2.2. Eligibility Criteria

The SPIDER eligibility framework for qualitative data (acronym for sample, phenomenon of interest, design, exclusion, and research type) [15], was used as a tool to include, or exclude articles of interest during an eligibility assessment as follows:

Sample (S): Acute (<6 weeks) to chronic (>3 months) of pain, as reassurance is an MSK best practice recommendation at any given time frame for an MSK condition [6]. Adults (≥18 years) were also selected because of the possible heterogeneity of outcomes, and the varied capacity to disseminate the impact of reassurance compared to children [16]. However, this recommendation is not supported by any existent systematic reviews that are specifically focused on MSK pain. Studies were excluded if the patients had MSK with another chronic or palliative condition.

Phenomena of Interest (PI): The studies involved observing at least two forms of the practitioner’s affective and cognitive reassurance [7,8], and how the patient-practitioner interaction had impacted the patient’s perception of their musculoskeletal pain. The patient-practitioner interaction could occur in any clinical setting (i.e., primary, or secondary), and the practitioner can be trained in any health-care profession (i.e., doctor, physiotherapist, nurse).

Design (D): The studies were required to utilize one of the following types of qualitative methodologies; types of phenomenology, types of grounded theory, different forms of action research, and narrative forms of research. Mixed methods studies were included once a qualitative analysis was conducted and once themes were identified.

Evaluation (E): The studies were required to include qualitative information pertaining to the impact of reassurance on the individual with MSK pain, in the form of consultation-exit outcomes.

Research Type (R): Qualitative studies or mixed methods studies that include a qualitative section.

Additional criteria: All the studies were published after the year 1979, corresponding to the year of Kessel’s [17] seminal article on doctor-patient reassurance. Studies that were reported in languages other than English were included if Google translate was able to provide a translation that was considered usable by both authors. Unpublished articles, conference proceedings, PHDs and theses were excluded.

### 2.3. Search Strategy

A systematic search strategy was conducted. The PRESS (Peer Review of Electronic Search Strategies) guidelines were utilized throughout record identification [18]. The articles used in this review were gathered from five electronic databases using database inception until December 2020, including Medline, AMED, CINAHL, Ergonomics, ProQuest Central. The following = key words; (1) “Reassurance”, (2) “Adult”, (3) “Qualitative Research”, (4) “Musculoskeletal Pain” were searched. In addition, Google Scholar and Science Direct were searched for the first 20 pages of hits from the main key words. The reference list of all the articles included in the research and all the of the review articles that have been identified were hand-searched [7,8,19].

### 2.4. Study Selection

The primary author undertook the main search, screened, and identified all potentially eligible studies.

### 2.5. Data Extraction

The data was extracted by the primary author using a table that identified key demographical information. Where an incomplete description of outcomes was identified, authors were contacted via email. If there was no response after two weeks, another email was sent, then a third and final email was delivered 1 week later. This data extraction process was also piloted for five articles before being utilized for all of the articles. No changes were made following the pilot.

### 2.6. Study Quality Assessment

The critical appraisal skills program (CASP) checklist was used to evaluate the rigor and trustworthiness of the qualitative studies [20]. The mixed methods appraisal tool (MMAT) [21] was used to assess the quality of the mixed methods designs.

### 2.7. Synthesis and Analysis of Results

The results section of the articles was thematically synthesized in accordance with the process of past reviews [8]. Three stages of analysis were conducted including; (a) open coding, which included an initial tabulation of results to provide comments for each paper. Each paper was considered to aggregate details regarding the impact of and the mechanisms relating to AR or CR. The definition of AR and CR were derived from the above definitions and used for categorization, (b) findings were then grouped by mind mapping both the mechanisms and the impact across the studies, (c) the findings were then condensed and considered for abstraction. In this process, the primary author was required to confirm the findings, search for similarities between themes and to remove themes where there was an overlap. The findings were then separated in relation to either a negative or a positive impact. The distinction for classification includes interactions that were associated with; (a) either negative (e.g., fear, anxiety, sadness) or positive emotions (e.g., calm, peaceful, joy), (b) either increased suffering, uncertainty and worry about the future or hope and possibility regarding the future, (c) a decrease in trust, a need to disengage, an inability to be understood, and a perceived need to limit or end the interaction or an increase in trust and a feeling of being understood. The supervising author acted as a critical confidante during this process.

### 2.8. Certainty Assessment

The GRADE Confidence in the Evidence from Reviews of Qualitative Research (GRADE CERQual) tool [22] was used to assess the certainty of the evidence. Using CERQual, the confidence of the evidence was assessed based on four areas: the methodological limitations, relevance, coherence, and the adequacy of data. This assessment allows for the grading of the data as high, moderate, low, or very low. A “high” CERQual in the data would indicate that the findings of the review are a rational demonstration of the phenomenon of interest [22]. Very low data will be defined as codes/subthemes supported by less than three studies, these codes will be largely excluded from the discussion and recommendations section due to an inadequate amount of data [22].

## 3. Results

### 3.1. Identification of Studies

**Figure 1:**
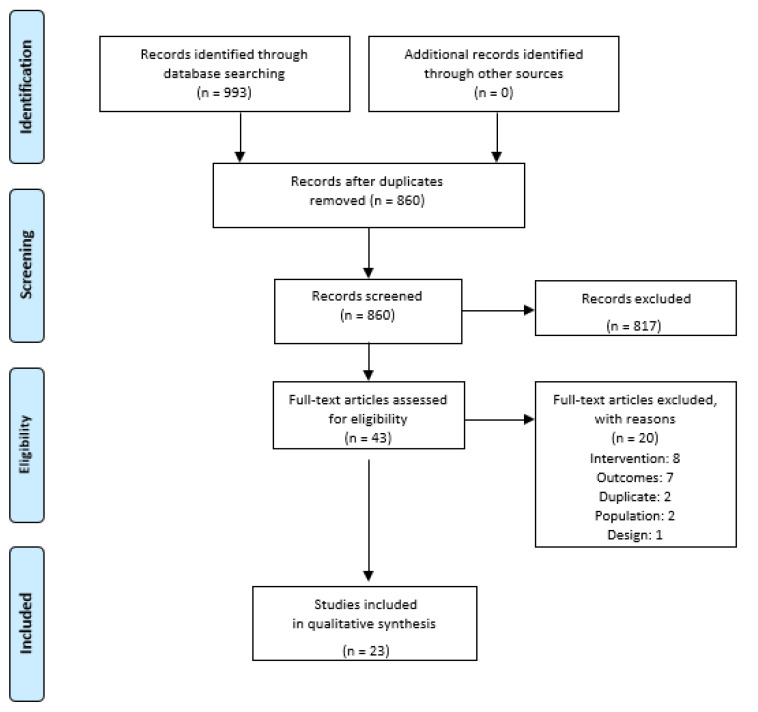
The PRISMA Flow Diagram.

A total of 993 studies were identified through database searching, of which 23 studies were [23,24,25,26,27,28,29,30,31,32,33,34,35,36,37,38,39,40,41,42,43,44,45] included for qualitative synthesis, with a total of 451 patients (271 were female, 180 male). See Supplementary Material File S1 Table S1 for details of the excluded articles and the demographics for the included studies and Supplementary Material File S1 Table S2 for detailed data extraction tables. The studies that have been included were published between 2000 and 2020 and conducted in the United Kingdom (*n* = 10), Norway (*n* = 4), Denmark (*n* = 3), New Zealand (*n* = 2), USA (*n* = 1), Netherlands (*n* = 2), and Ireland (*n* = 1). A range of practitioners were interviewed, although most of the practitioners included were physicians (*n* = 48). The number of other allied health care professionals could not be obtained from the manuscripts. A total of 8 studies [23,24,25,26,30,32,34,35] focused on physician encounters. A total of seven studies focused on physiotherapist interactions within interventions [27,31,37,39,44,45]. As part of the thematic results, we identified occasions in which at least three studies for either a physician or physiotherapist are found to represent a theme or sub-theme. A range of diagnoses were included and the most frequent (*n* = 8) was for back pain or low back pain (LBP), a further four related to rheumatic patients or patients who were identified as experiencing chronic pain. See Figure 1 for a summary of the study identification.

### 3.2. Quality Assessment

The CASP quality assessment findings provide that all the studies establish clear aims, appropriate qualitative methods, a clear statement of findings, and contribute to our understanding on the impact of reassurance for adults with MSK pain. However, the quality of the research design, recruitment strategy, the data collection method, the rigor of the data analysis, ethical issues and practical applications varied. Several studies failed to adequately consider the relationship between researcher and participants. The MMAT quality assessment findings identified that all studies establish clear research questions, the data collected in the studies addresses the research questions, that there is adequate rationale for the use of mixed methods, that different components of the study are integrated to answer the research question, the outputs of integrating qualitative and quantitative components and that any divergencies and inconsistencies between quantitative and qualitative results are addressed. Refer to Supplementary Material File S1 Table S3 for a summary of the methodology’s quality assessment.

### 3.3. Identification of Themes

**Figure 2:**
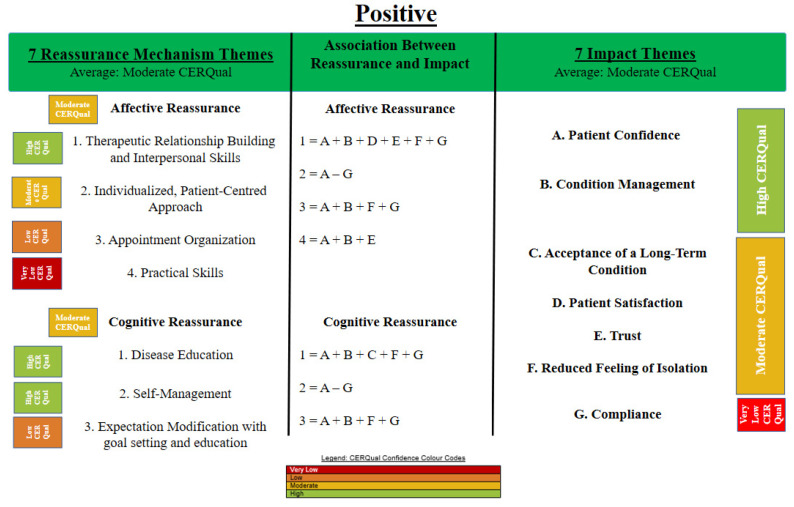
CERQual summary of thematic findings displaying the positive impact of reassurance and mechanisms. Note: View this figure from left to right. The left of the figure identifies mechanisms within interactions. The association between mechanism and impact is illustrated at the center of the figure and identifies how mechanism relate to multiple outcomes identified on the right of the figure.

**Figure 3:**
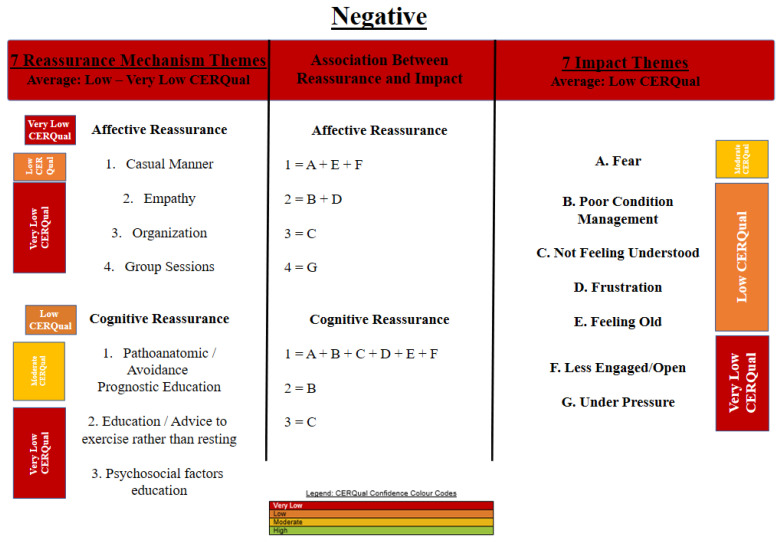
CERQual summary of thematic findings displaying the negative impact of reassurance and mechanisms. Note: View this figure from left to right. The left of the figure identifies mechanisms within interactions. The association between mechanism and impact is illustrated in the center of the figure which identifies how mechanism relate to multiple outcomes identified on the right of the figure.

An initial thematic synthesis on the impact of reassurance identified both the positive and negative impacts of reassurance and identified its associated mechanisms. Figure 2 and Figure 3 provide details of the resultant themes and mechanisms. The average CERQual assessment for the positive impact and mechanism themes (most often identified as moderate CERQual evidence) were stronger than the evidence for the negative impact and mechanism themes (most often identified as low to very low CERQual evidence). See Supplementary Material File S1 Tables S4–S9 for a further detailed explanation of the tables. These tables provide a detailed rationale for the CerQual assessment rating.

Figure 2 displays three critical outcomes. On the right of Figure 2 the impact of positive interactions can be identified. This also includes a CerQual evidence rating. This highlights the most robust evidence in support of reassurance impacting patient confidence and condition management. On the left of Figure 2, the mechanisms that explain the impact are presented according to the type of reassurance and are organised according to their CerQual evidence rating. High CerQual ratings were found for therapeutic relationship building and interpersonal skills with AR and also for disease education and self-management in CR. A CerQual rating for AR and CR can be more generally observed. In the central section of Figure 2 the mechanism that have performed a role in the different impact outcomes have been allocated based on the type of reassurance. The following AR mechanisms identified that: (b) Therapeutic relationship building and interpersonal skills are associated with all of the positive impact themes. (b) An individualized, patient-centered approach was associated with improving patient confidence, condition management, acceptance of a long-term condition and a reduced feeling of isolation. (c) Appointment organization was associated with all the positive impact themes except for a reduced feeling of isolation. (d) Practical skills were associated with patient confidence, condition management and trust.

The following CR mechanisms identified that: (a) Disease education was associated with all of the positive impacts except for patient satisfaction and trust. (b) Self-management was associated with all of the positive impact themes and (c) expectation modification using goal setting and education was associated with confidence, condition management, reduced feeling of isolation and compliance. Reassurance mechanisms were categorized into affective and cognitive types of reassurance.

Figure 3 displays three critical outcomes. On the right of Figure 3 the impact of negative interactions is considered, includes their CerQual rating. This displays the highest level of support for the negative impact of fear. On the left of Figure 3 the mechanisms that explain the impact are presented according to the type of reassurance and each is allocated a rating. You can also observe a CerQual rating for AR and CR more generally. The most supported mechanisms included pathoanatomic/avoidance of prognostic education which received a moderate CerQual rating. In the middle section of Figure 3 the mechanism that performs a role in the different impact outcomes are categorized by the type of reassurance. The AR mechanisms identified the following: (a) A practitioner’s casual manner was associated with a patient’s fear, frustration and the patient being less engaged/open. (b) A patient could perceive a lack of empathy by listening to other people’s stories (that were perceived as unachievable) in a pain therapy session. The stories were often associated with poor condition management and frustration. (c) Organizational issues, for instance case appointment cancellation was associated with a patient not feeling understood, and (d) group sessions, involving exercise could be associated with placing the patient under pressure due to patients being unable to perform as well as the rest of their group members.

The CR mechanisms identified that; (a), pathoanatomic, avoidance prognostic education may be associated with poor condition management, not feeling understood, frustration, feeling old and being less engaged and open. (b) Education and advice to exercise rather than resting could be associated with poor condition management, and (c) lastly, an education on psychosocial factors was associated with the patient not feeling understood.

### 3.4. The Impact and Associated Mechanism with at Least Moderate CerQual Evidence

The results of the current synthesis assisted in identifying the relevant themes. Table 1 provides information on how the themes and sub-themes were deconstructed, possessing at least a moderate CerQual rating.

#### 3.4.1. The Positive Impact

There are seven positive impact themes, each with multiple subthemes, some of with poor data.

Theme 1: Patient Confidence

Patient confidence is defined as the patient’s perceived ability to engage in health promoting actions [46]. The patient confidence theme was classified as having high CERQual evidence. It contained seven subthemes.

Sub Theme 1: Enablement/Motivation

This sub-theme was defined as a patient’s willingness to pursue the management of their condition and possessed the highest quality of evidence for the patient confidence theme. Ten studies support this code [28,29,31,35,37,38,39,40,42,43]. This included a total of four studies that reported on physiotherapy interactions [31,37,39,42].

Sub Theme 2: Self-Confidence

This sub-theme was defined as patients personally being more hopeful with regard to the present and future in terms of living with their condition. Five studies supported this sub-theme [27,29,32,38,41].

Sub Theme 3: Patient participation

This sub-theme concerned the willingness of patients to be honest about their condition and whether they adhered to the advice and education provided. This sub-theme was supported by three studies [20,39,40] and mainly focused on the value of rapport via a therapeutic relationship and provided the patient with individualized education.

Moreover, the remaining 4 subthemes that contributed to the main patient confidence impact of reassurance theme had thin data.

Theme 2: Condition Management

This sub-theme was defined as improving the quality of life for individuals by minimizing the effects of a health condition [47]. The improved condition management theme, as an impact of reassurance, was classified as possessing high CERQual evidence. This theme had seven subthemes.

Sub Theme 1: Self-Efficacy

This sub-theme was defined as a patient’s knowledge and perceived ability to control their condition’s symptoms. This sub-theme was the most supported positive impact for improving a patient’s condition management. This subtheme was supported by seven studies [31,35,36,37,40,43,44]. This included a total of three studies reporting on physiotherapy interactions [31,37,44].

Sub Theme 2: Feeling understood

This sub-theme relates to patients feeling better understood with regard to their experience of managing their condition, through a therapeutic relationship with emotional intelligence, validating the patient’s issues with objective tests, and providing individualized treatment and education strategies. Seven studies identified positive impact within patients, who felt more understood and supported by their practitioner [24,34,36,38,40,43,45].

Sub Theme 3: Belief Change—Importance of exercise

This sub-theme was defined as patient learning and belief in the importance of exercise to improve the state of their condition. Four studies found a positive impact from cognitive reassurance, including education, encouraging self-management strategies and affective reassurance through a patient-centered approach to care. This type of reassurance resulted in a belief change on the importance of exercise [38,39,41,42].

Sub Theme 4: Behaviour Change—Physical Activity/Exercise/Movement

This sub-theme was related to the increasing physical activity of patients, alongside their exercise and movement levels. Four studies found that the combination of all types of CR and AR resulted in behaviour change towards physical activity [33,36,37].

Theme 3: Acceptance of a Long-Term Condition

This theme related to patients realizing that pain does not mean that their functioning and activity must stop [48]. The provision of AR, with a focus on the patient’s priorities, future self-management advice and education regarding why the patient experienced pain, was central to this. Three studies with a moderate CERQual evidence score demonstrated that patients had a more positive outlook of their issue as a long-term condition if acceptance could be considered [23,29,42].

Theme 4: Patient Satisfaction

This theme was defined as a patient’s positive evaluation of their care, which was explored in three studies with moderate CERQual evidence scores, when CR was provided, with affection [28,31,34]. The data regarding satisfaction was also associated with practitioners considering the patient’s thoughts, preferences, and feelings, and providing positive feedback to the patient, as well as being friendly and remembering facts about the patient [31,34].

Theme 5: Trust

This theme was defined as the patient’s trust in the practitioner to improve their condition. A total of four studies of moderate CERQual evidence were identified. This theme comprised of three subthemes, including the patient trusting the practitioner, the patient not feeling judged and the patient being more open and honest [35,36,40,45].

Theme 6: Reduced Feeling of Isolation

This theme was related to the diminished level of isolation felt by patients = after receiving support, as well as their experience of motivation and learning during group discussion from peers who were also experiencing MSK rehabilitation. This theme was supported by three studies [39,41,44].

Theme 7: Compliance

This theme had thin data and has been placed into the Supplementary Material File S1 paragraph S1.

#### 3.4.2. The Positive Mechanisms

##### Affective Reassurance

Theme 1: Therapeutic relationship building and interpersonal skills

This theme was defined as the of a rapport and a working alliance to enhance patient motivation and ownership [45]. Fourteen studies contribute to theme 1, with no or very minor CERQual concerns, except a moderate data concern as a result of thin data in nine of the 11 codes, resulting in a high CERQual evidence [24,25,27,28,29,31,33,36,37,39,40,43,44,45]. This included a total of six studies that reported on physiotherapy interactions [27,31,37,39,44,45].

Subtheme 1: Interpersonal skills and relationship building

This theme encompasses mutual support, activating and partnering with the patient, open, honest and trusted listening and shared decision making had the highest adequacy of data within theme 1, with nine studies supporting this sub-theme [24,25,31,36,37,39,43,44,45]. This included a total of five studies reporting on physiotherapy interactions [31,37,39,44,45].

Subtheme 2: Narratives of other MSK patients, sharing their insights and experiences, had the second greatest support within theme 1, with 3 studies supporting this sub-theme [28,39,44].

Theme 2: Individualized, patient-centered approach

Theme 2 encompasses a biopsychosocial perspective, represented by the following characteristics: seeing the patient-as-person, sharing power and responsibility in interactions, and upholding the therapeutic alliance [49]. This theme can be considered as a recommendation as well, due to moderate CERQual evidence [23,25,28,34,39]. However, none of the codes for theme 2 prove to be significant findings due to thin data. Themes 3–4 had low to very low CERQual confidence in evidence.

##### Cognitive Reassurance

Theme 1 and 2: Disease Education and Self-Management, had a high CERQual confidence in evidence.

Theme 1: Disease Education

There were three main defining subthemes that represented disease education. These included: (a) information about the disease and its prognosis, diagnosis/testing, aetiology, exercise and treatment [25,28,31,34,35,36,38,39,40,43,44]. This included a total of three studies reporting on doctor interactions [25,34,35] and three considered physiotherapist interactions [31,39,44]. (b) An interaction that identifies a positive outlook or can be perceived in a positive light (e.g., pain does not always equal damage) [25,37,41] and (c) asking patients to ask questions in return [38,42,43]. These were the only subthemes out of the 13 that were supported with adequate data.

Theme 2: Self-Management

There was a total of two defining subthemes (1) convenient and fun self-help exercises involving functional activities and how to perform them [27,31,37,38,40,44,45]. This included a total of five studies reporting on physiotherapist interactions [27,31,37,44,45]. (2) Self-management combinations (exercise, pain relief) [23,24,28,35,39], including a total of three studies reporting on doctor interactions [23,24,35], which had adequate data. The third and last theme, expectation modification with goal setting and education [31,40], had a low CERQual confidence in evidence.

#### 3.4.3. The Negative Impact

Theme 1: Fear

This theme was defined as an intense/overwhelming worry which led to modified thoughts and behaviors that are likely a result from a low perceived confidence or an inability to change a negative pain related outcome. Fear was most often caused by the introduction of doubt, uncertainty and an emphasis on potentially negative pain related outcomes, compounded by paternalistic interactions that lacked affection and/or sensitivity in information delivery. Three studies found that patients experienced the greatest fear towards the future of their condition from unaffectionate cognitive reassurance [30,32,36]. The cognitive reassurance component of these studies involved educating the patient about the prognosis and aetiology of the condition using pathoanatomic models. However, the lack of affection resulted in the patients interpreting the language in a way that induced worry regarding the seriousness of their condition, despite having a common non-sinister MSK condition. Examples of language choices included: intervertebral disc degeneration, intervertebral disc donut and jam analogy and an emphasis on the early stage despite patients already experiencing anxiety, causing them to avoid activities to protect their backs using pathoanatomic explanations.

Theme 2: Poor Condition Management

The remaining 6 themes: (1) poor condition management, (2) not feeling understood, (3) frustration, (4) feeling old, (5) less engaged, honest and open, (6) under pressure, had a low to very low CERQual. Due to the thin and poor-quality data, details regarding these themes can be found in the Supplementary Material File S1 paragraph S1.

#### 3.4.4. The Negative Mechanisms

There were four themes within affective reassurance, whereas cognitive reassurance had three mechanism themes. The themes are identified below.

##### Affective Reassurance

Theme 1 (causal manner) had a low CERQual confidence in evidence [32,36]. Furthermore, the other themes, (2) empathy [44] (3) organization [43] and (4) group sessions [39] had a very low CERQual confidence in evidence. The low to very low CERQual scores means all of these reassurance mechanisms are inappropriate to develop a firm understanding of these mechanisms.

##### Cognitive Reassurance

Theme 1, pathoanatomic and avoidance prognostic education, defined as education to avoid physical activity due to future pathological deterioration has a moderate CERQual confidence in evidence [30,32,35]. While the remaining 2 reassurance mechanisms (2) education/advice to exercise as a treatment rather than resting [38] and (3) psychosocial factors education, regarding the influence on pain [43], both have a very low CERQual confidence in evidence. This provides a case for pathoanatomic and avoidance prognostic education with adequate CERQual.

## 4. Discussion

The current review was able to consider the impact and mechanisms associated with reassurance for patients with MSK pain, across different clinical settings. The findings of this review demonstrate that there are primarily 2 positive impact themes of reassurance that possessed a high CERQual score including patient confidence and condition management. The reasons for this impact are likely related to processes associated with patient education and the promotion of self-management. Due to the low CerQual evidence scores the remaining themes regarding impact and mechanism require further evidence to be established. The negative impact and mechanisms had weaker evidence. The greatest level of confidence could be placed in the impact of fear from a negative encounter which appeared to result from a lack of explanation about the condition and not providing prognostic education. This evidence for the negative themes was associated with a moderate CERQual.

### 4.1. Value of Effective Cognitive Reassurance

CR skills are required to help change pain beliefs from biomechanical and pathoanatomical discourses to more complex biopsychosocial factors, as part of person-centered communication skills in MSK healthcare professional’s training programs [50]. So, this review highlights the importance of discourse and semantics during CR, to prevent catastrophizing and to confront patient’s stressors, to minimize long-term cortisol secretion and to encourage positive patient outcomes [51]. These maladaptive stressors can be supported by providing an education that involves pain neuroscience education, cognitive behavioral therapy and cognitive functional therapy and self-management strategies [52].

### 4.2. Therapeutic Relationship and Person-Centered Care

The current results suggest that a more individualized, patient-centered approach to care can improve patient confidence. This can be formed by building a therapeutic relationship with the patient [37,53]. Multiple guidance documents are available that identify how this can be achieved, for instance, policy documents have identified stage approaches to care [54,55]. Furthermore, via health promotion and prevention programs, as well as by measuring the performance of person-centered care a reassuring patient-practitioner interaction will be maintained in organizations and will encourage the improvement of best practice [56].

### 4.3. Interpersonal Skills and Relationship Building

Past review-based research has supported the value of relationship building skills [7,19]. In addition to this, the qualitative systematic review performed by Kinney et al. [57] details the importance of trust and open communication with the patient to improve therapeutic alliance, which is associated with positive patient outcomes. The current findings further this by identifying specific interpersonal relationship building skills that aid the interaction. These skills including being open and honest with the patient, shared decision making, trusted listening and activating which are an integral part of creating a therapeutic relationship and have a positive impact on the patient. Another systematic review by Shay and Lafata [58] also found that shared decision making resulted in a positive impact on the patient’s understanding, satisfaction and trust in the health care professional, which were also themes with a moderate CERQual in this review.

### 4.4. Patient Stories

This review also found adequate data on the positive impact of storytelling or peer sharing from other MSK patients. This finding is validated by the systematic review from Toye et al. [59], who found that patients that were part of a community of other individuals suffering from the same pain experiences, and able to discuss their pain with people, benefitted patients by counselling them to positively move forward with their pain. This positive impact finding by Toye et al. [59] was also corroborated by this review, as stories made patients more confident and enabled them to improve the present and future state of their condition.

### 4.5. Patient Confidence

A systematic review and meta-analysis by O’Halloran et al. [60] found that motivational interviewing, a type of CR and AR that can be categorized as education and created a therapeutic relationship with the patient, leads to improvements in physical activity for chronic pain patients. Furthermore, another systematic review and meta-analysis conducted by Alperstein and Sharpe [61], found that motivational interviewing increased adherence to chronic pain treatment. Similarly, this review found that CR and AR improve patient confidence for participating in health promoting behaviour. This includes an increased ability and motivation to follow advice and education from healthcare professionals. Interestingly, Pincus et al. [8] found that CR has a hypothesized predictor towards enhanced self-efficacy, which was validated in this review with a high CERQual.

### 4.6. Feeling Understood

Past review evidence [8] found that AR and CR is a possible predictor for patients feeling supported, suggesting that patients feel that healthcare professionals understand what the patient is going through. Similarly, this review also found that AR and CR made patients feel more understood, with a high CERQual. Additionally, Pincus et al., [8] deduced that AR and CR improved patient satisfaction and the trust in the practitioner, which was also found in this review with moderate CERQual.

### 4.7. Fear Inducing Cognitive Reassurance and Maladaptive Beliefs

The patient’s distress from fear inducing cognitive reassurance is suggested by a recent systematic review from O’Keefe et al. [7], who highlights the importance of a physical therapist’s interpersonal and communication skills to reduce the risk of a negative impact on the patient. However, the negative impacts of reassurance were not highlighted. They also were not identified in the recent quantitative reviews e.g. [8,19]. This review has deduced that an increase in the patient’s fear can be associated with poor consideration to providing education on the condition. This finding is valuable, as it highlights the importance of avoiding the use of fear inducing education, such education can perpetuate pain related fear cognitions and fear-avoidance beliefs [62]. Clinicians need to recognize that a patient’s distress and fear may be derived from previous maladaptive beliefs inherited from healthcare professional’s education and explanations [63]. These beliefs can perpetuate their tendency of catastrophizing pain [64]. Catastrophizing may result in the intensification of cortisol secretion, sensitizing a stress response, perpetuating pain in MSK conditions by reducing patient self-efficacy and negatively impacting their outcomes [63]. Education needs to be reconsidered and corrected with positive reassurance mechanisms.

## 5. Limitations

There were limitations in the search strategies, for instance, grey literature and specific databases such as PEDro were not included in this review, which reduces the sample size of an individual’s lived experiences and a generalizable impact from reassurance. This review was conducted independently, increasing the risk of a confirmation bias [65]. Methodological limitations may exist resulting from our choice of the critical appraisal tool. Specific types of therapy that do involve reassurance, for instance cognitive behavioral therapy and acceptance commitment therapy were not included. The complex patient context was also not considered, including the patient’s sociocultural, economical and ethnic contexts, which may alter the reasoning for using specific types of reassurance and the impact it may have on these different patient populations. In this review, the definition of AR and CR derived Pincus et al. [8] was quite broad, often making it challenging to classify interactions as AR. This may have reduced the number of studies that deduce the impact of an AR mechanism. There should be a focus on the contradicting impacts of CR, as this review has found. A review and meta-analysis of mixed method studies can help to identify direct causes or relationships between mechanism of reassurance and the specific impact on the individual, by addressing contradictions between qualitative and quantitative results [66].

## 6. Implications

The current results primarily included studies that considered interactions by physicians or physiotherapists. The strongest association with a positive impact and mechanisms was found within the studies that included physiotherapists. This was likely due to the interventional nature of these studies. However, the findings are useful and could be applied by other healthcare practitioners interacting with MSK pain patients. The current results suggest a combination of AR, encompassing therapeutic relationship building, interpersonal skills and an individualized patient-centered approach. It is also noted that disease education and self-management are required for a more positive impact. This is important as these mechanisms of reassurance reliably improve patient confidence and condition management. Healthcare professionals can also consider avoiding fear inducing education v pathoanatomic avoidance and prognostic education.

There is a need for a greater assessment of the patient’s worries, concerns, feelings and beliefs to address the possible maladaptive beliefs a patient may have, and attempt to shift a patient’s maladaptive pain beliefs [32]. In post consultation documentation, the type of reassurance a practitioner provides to a patient should be clearly outlined. Having a clearer understanding of what types of reassurance has positively or negatively impacted the patient can aid in the clinical decision making for what type or mechanism of reassurance a practitioner can use during a patient interaction.

Clinical practice guidelines need to recommend the use of AR with an avoidance of fear inducing CR, as CR is widely recommended, however there are no specific recommendations for the use of AR.

## 7. Conclusions

The current review highlights the positive impact and mechanisms of AR and CR during interactions with people who have MSK pain conditions. Specific positive impact and mechanisms were identified during physiotherapist-patient interactions and doctor-patient interactions. There is less clarity and confidence in evidence around the negative impact and the mechanisms of reassurance. However, it is clear that MSK practitioners should avoid using of pathoanatomic and avoidance prognostic education during interactions.

## Figures and Tables

**Table 1:** Showing the impact and mechanisms associated with reassurance with at least moderate CerQual evidence.

Impact Theme	Impact Sub-Theme	Mechanism Theme	Mechanism Sub-Theme
**Positive impact**			
**Patient Confidence** **Definition: The patient’s perceived ability to engage in health promoting actions**	Enablement motivation	AR: Therapeutic relationship building and interpersonal skills	AR: Interpersonal skills and relationship building
	Self-confidence		AR: Individualised, patient-centered approach
	Patient participation		
**Condition management** **Definition: Improving quality of life for individuals by minimizing the effects of a health condition**	Self-efficacy		
	Feeling understood		
	Belief change		
	Behaviour change		
**Acceptance of a long-term condition** **Definition: Patients realizing that pain does not mean that function and activity needs to stop**			
**Patient Satisfaction** **Definition: Patient’s positive evaluation of their care**			
**Trust** **Definition: the patient’s trust in the practitioner to improve their condition**			
**Disease education** **Definition: Education that involved the following qualities; (a) information about the disease and prognosis, diagnosis, testing and treatment, (b) identification of positive information regarding the pain (c) asking patients to ask questions.**	Information about the disease and prognosis		
	Self-management		
**Negative impact**			
**Fear** **Definition: An intense/overwhelming worry which cause modified thoughts and behaviors which are likely a result from a low perceived confidence or ability to change a negative pain related outcome.**		Pathoanatomic and avoidance of prognostic education	

## Data Availability

An analysis can be found in the supplementary file.

## Supplementary Materials

The following are available online at https://www.mdpi.com/article/10.3390/bs11110150/s1. Table S1: Excluded articles; Table S2 data collection and extraction; Table S3 quality assessment of included studies using CASP qualitative checklist and MMAT checklist; Table S4 the impact of reassurance styles; Table S5 CERQual summary of qualitative findings—positive impact; Table S6 CERQual summary of qualitative findings—negative impact; Table S7 CERQual summary of qualitative findings—negative impact; Table S8 CERQual summary of qualitative findings—positive impact; Table S9 CERQual summary of qualitative findings—negative impact; Paragraph S1 additional themes, subthemes and codes within the data.
