# Supplementary material for: The Impact of Reassurance on Musculoskeletal (MSK) Pain: A Qualitative Review
Source: Behav Sci (Basel). 2021 Oct 30;11(11):150. doi: 10.3390/bs11110150 (PMC8615035; doi:10.3390/bs11110150)
Supplement: Supplementary file 1 [file behavsci-11-00150-s001.zip › behavsci-1426114-supplementary.pdf]

Table S1: Excluded Articles

1	Paskins et al., 2015<br><br>The Identity Crisis of Osteoarthritis in General Practice: A Qualitative Study Using Video-Stimulated Recall<br><br>https://pubmed.ncbi.nlm.nih.gov/26553893/<br><br>Found from: Similar articles for PMID: 10688559	No affective reassurance.
2	Giroldi et al., 2014<br><br>"Doctor, please tell me it's nothing serious": an exploration of patients' worrying and reassuring cognitions using stimulated recall interviews.<br>https://pubmed.ncbi.nlm.nih.gov/24762333/<br><br>Found from: Similar articles for PMID: 10688559	Limited affective reassurance and limited number of patients with msk conditions.
3	Kongsted et al., 2018<br><br>Feasibility of the consultation-based reassurance questionnaire in Danish chiropractic practice<br>https://www.ncbi.nlm.nih.gov/pmc/articles/PMC6116376/<br><br>Found from Similar articles for PMID: 30933288	Does not investigate the impact of reassurance on the patient
4	Kidd, Bond and Bell, 2011<br><br>Patients' perspectives of patient-centredness as important in musculoskeletal physiotherapy interactions: a qualitative study<br><br>https://www.sciencedirect.com/science/article/pii/S0031940610000957	Duplicate from O'Keefe

5	Dean et al., 2009<br><br>Managing time: An interpretative phenomenological analysis of patients' and physiotherapists' perceptions of adherence to therapeutic exercise for low back pain<br><br>https://www.tandfonline.com/doi/pdf/10.1080/09638200500030449?needAccess=true	The interviews lacked questioning around the reassurance aspect of the consultations
6	Vaillancourt et al., 2017<br>Patients' Perspectives on Outcomes of Care After Discharge From the Emergency Department: A Qualitative Study<br><br>https://www.sciencedirect.com/science/article/pii/S0196064417306704	Limited number of msk patients
7	Harman et al., 2011<br><br>Client Education: Communicative Interaction between Physiotherapists and Clients with Subacute Low Back Pain in Private Practice<br><br>https://www.utpjournals.press/doi/pdf/10.3138/ptc.2009-52P	Physiotherapists interviewed
8	Ward et al., 2007<br><br>Patient priorities of care in rheumatology outpatient clinics: a qualitative study<br>https://pubmed.ncbi.nlm.nih.gov/17657707/<br><br>Found from: Similar articles for PMID: 10688559	No mention of how cognitive reassurance impacted the patient
9	Zanini et al., 2016<br><br>Enhancing clinical decisions about care through a pre-consultation sheet that captures patients' views on their health conditions and treatments: A qualitative study in the field of chronic pain<br>https://www.sciencedirect.com/science/article/pii/S073839911530152X?via%3Dihub<br>Found from: Similar articles for PMID: 10688559	outcomes
10	Tjong et al., 2015	No mention of cognitive reassurance

	A Qualitative Investigation of Return to Sport After Arthroscopic Bankart Repair: Beyond Stability<br><br>https://journals.sagepub.com/doi/10.1177/0363546515590222?url_ver=Z39.88-2003&rfr_id=ori:rid:crossref.org&rfr_dat=cr_pub%20%200pubmed	
11	Grahn, Stigmar and Ekdahl<br><br>Motivation for change in patients with prolonged musculoskeletal disorders: a qualitative two-year follow-up study<br><br>https://onlinelibrary.wiley.com/doi/abs/10.1002/pri.164?sid=nlm%3Apubmed	No mention of cognitive reassurance
12	Petersen and la Cour, 2016<br><br>Mindfulness--What Works for Whom? Referral, Feasibility, and User Perspectives Regarding Patients with Mixed Chronic Pain<br><br>https://pubmed.ncbi.nlm.nih.gov/26986534/	No mention of cognitive reassurance
13	Ross et al., 2014<br><br>An effective coaching intervention for people with low recovery expectations and low back pain: A content analysis<br><br>https://content.iospress.com/articles/journal-of-back-and-musculoskeletal-rehabilitation/bmr00424	Does not capture qualitative data on consultation-exit outcomes
14	Lyons et al., 2013<br><br>Perspectives of older adults on co-management of low back pain by doctors of chiropractic and family medicine physicians: a focus group study<br>https://pubmed.ncbi.nlm.nih.gov/24040970/	Does not capture patient's outcomes

15	Nagl, Ullrich and Farin, 2013<br><br>[Comprehensibility of patient education in orthopaedic rehabilitation: a qualitative study on patients and providers]<br><br>https://pubmed.ncbi.nlm.nih.gov/22753052/	Lacks focus on the impact of a type of reassuring intervention on the patient’s outcomes (fear, self-efficacy, disability, understanding, confidence, self-esteem, anxiety, depression), looks at the comprehensibility.
16	Hendry et al., 2006<br><br>Why should we exercise when our knees hurt? A qualitative study of primary care patients with osteoarthritis of the knee<br><br>https://pubmed.ncbi.nlm.nih.gov/16731544/	No mention of affective reassurance, just “advice”, “education”, no “rapport”, “empathy”, “compassion”, “common understanding” explicitly provided during a patient-practitioner interaction, just during referrals to gyms.
17	Williams and Graham, 2012	No mention of affective types of reassurance

	'My feet: visible, but ignored . . .' A qualitative study of foot care for people with rheumatoid arthritis<br>https://pubmed.ncbi.nlm.nih.gov/22275462/	
18	Miller and Gabbay<br><br>Motivating patients with shoulder and back pain to self-care: can a videotape of exercise support physiotherapy?<br><br>https://pubmed.ncbi.nlm.nih.gov/19627683/	No affective reassurance
19	Shaw et al., 2011<br><br>https://www.jabfm.org/content/24/1/16.long	no qualitative section
20	Holt et al., 2018<br><br>https://journals.lww.com/clinicalpain/fulltext/2018/04000/testing_a_model_of_consultation_based_reassurance.7.aspx	No qualitative section

Table S2: Data collection and extraction

<table>
<tr><th rowspan="2">#</th><th rowspan="2">Source</th><th rowspan="2">Sample</th><th colspan="2">Phenomenon of Interest</th><th rowspan="2">Design</th><th rowspan="2">Evaluation</th></tr>
<tr><th>Affective Reassurance (4)</th><th>Cognitive Reassurance (2)</th></tr>
<tr><td>1</td><td>Dures et al., 2016</td><td>Country
UK

Participants
19 Patients
74% Males 26% Females
25 – 75 Years Old
16 Chronic Rheumatoid Arthritis
3 Chronic Psoriatic Arthritis

Practitioners
2 Rheumatology Consultants, 1 Nurse Specialist, 1 Occupational Therapist</td><td>Flexible Appointments
Routine consultations as a way of support

Patient-Centred Approach
Appreciating the holistic (physical, social and emotional) impact of IA (Inflammatory Arthritis).

Focusing on patient’s priorities

The use of open, patient-centred communication styles, rather than didactic approaches.</td><td>Self-Management
Pacing education specific to the patient’s priorities

Self-management advice specific to the patient’s issues</td><td>Inductive thematic analysis by reading the transcript of semi-structured interviews.

Interviews were designed by the research team, including patient partners, rheumatology and psychology clinicians and qualitative methodologists</td><td>Positive
Patient Participation
Increased confidence to be proactive in the consultation.

Self-Confidence
Valuing their own experience and expertise, which improved their ability to deal with the impact of inflammatory arthritis.

Acceptance of a long-term condition
Greater acceptance of a long-term condition.</td></tr>
</table>

<table>
<tr><th rowspan="2">#</th><th rowspan="2">Source</th><th rowspan="2">Sample</th><th colspan="2">Phenomenon of Interest</th><th rowspan="2">Design</th><th rowspan="2">Evaluation</th></tr>
<tr><th>Affective Reassurance
(9)</th><th>Cognitive Reassurance
(4)</th></tr>
<tr><td>2</td><td>Vinall-Collier, Madill, and Firth, 2016</td><td><u>Country</u>
UK

<u>Participants</u>
15 Patients
27% Male / 73% Female
49-71 Years Old
Acute-Chronic Rheumatoid Arthritis (RA) or
Unconfirmed RA (Specific Statistics Unclear)

<u>Practitioners</u>
N = 18
9 Physicians
9 Nurse Specialists</td><td><u>Interpersonal Skills</u>
Building a relationship (personal remarks, laughs, approval, compliment, agreement, empathy, concern/worry, disagreement/criticism, legitimizing statements)

Activating and partnering</td><td><u>Education</u>
Patient education and counselling

Giving therapeutic information

Giving information about lifestyle.</td><td>Roter's Interactional Analysis System (RIAS) was used to code data. 31 semi-structured interviews with 16 practitioners and 15 patients within 24-hour consultations were thematically analysed.</td><td>Positive
<u>Peace of Mind</u>
Continuity of practitioner benefited the establishment of a relationship, providing the patient with emotional support.

<u>Patient Participation</u>
Improved familiarity with a patient's medical history, personal circumstances, which improved patient participation.</td></tr>
<tr><td>3</td><td>Haugli, Strand and Finset, 2004

https://www.sciencedirect.com/science/article/pii/S0738399103000235?via%3Dihub</td><td><u>Country</u>
Norway

<u>Participants</u>
26 Patients
25% Males / 75% Females
20-80 Years Old
12 Chronic Inflammatory MSK condition (e.g. RA)
14 Chronic Non-Inflammatory Wide-Spread Pain Condition (e.g. Fibromyalgia)

<u>Practitioners</u></td><td>(8)
<u>Empathy</u>
Taking the perspective of the patient

<u>Making the patient feeling cared for and understood</u>
Being seen as an individual and not as a disease entity

Giving information of the disease with an open manner, neither too positive or negative

Being believed

Being taken seriously</td><td>(3)
<u>Education</u>
Giving adequate information about the disease and prognosis

Giving a diagnosis

Shared understanding of the aetiology and treatment</td><td>Focus group interview with a semi-structured interview guide.

Qualitative analytical process</td><td>Positive
<u>Feeling of Security</u>
The feeling of security

<u>Pain Reduction</u>
Pain reduction – after being believed</td></tr>
</table>

		Primary care physician, Local Rheumatological Outpatient department or ward	Reciprocal trust through honesty<br><br>Flexible Appointments<br>Availability – being able to contact the physician when symptoms exacerbate.<br><br>Enough dialogue time for continuity of care			

#	Source	Sample	Phenomenon of Interest		Design	Evaluation
			Affective Reassurance	Cognitive Reassurance		
4	Saunders et al., 2020	Country<br>UK<br><br>Participants<br>20 Patients<br>50% Male / 50% Female<br>28-86 Years Old<br>Chronic Sciatica<br><br>Practitioners<br>N = 20; 7 Spinal Physiotherapists; 9 GPs; 4 Spinal Surgeons.	(1)<br>Making the patient feeling cared for<br>Short waiting times for an MRI scan (5 days)	(1)<br>Scan Education<br>Interpretation of a MRI scan, showing where a disc bulge is, and there is nothing sinister	Semi-structured interviews	**Positive**<br>Feeling of security<br>Earlier reassurance in understanding the cause of their pain and assuring that there is no serious underlying pathology. "I know what I'm coping with and I just feel easier now. That's 90% of the battle really, that I know it's nothing too sinister.

#	Source	Sample	Phenomenon of Interest		Design	Evaluation
			Affective Reassurance	Cognitive Reassurance		
5	Kidd, Bond, and Bell, 2011	Country<br>New Zealand<br><br>Participants<br>N = 8<br>50% Females / 50% Males<br>20 – 68 years old<br>Recently received physiotherapy at musculoskeletal outpatient physiotherapy.	(7)<br>Rapport<br>Ability to communicate<br>Confidence<br>Knowledge and Professionalism<br><br>Empathy<br>Understanding of people and an ability to relate<br><br>Understood<br>Transparency of progress and outcome<br><br>Making the patient feeling cared for<br>Encouragement	(2)<br>Education and Explanation<br>Reassurance about the pain being ok.<br><br>Education and instructions for self-help and exercise.	Qualitative study using semi-structured interviews to explore patients' judgements of patient-centered physiotherapy. Grounded theory was used to determine common themes among the interviews and develop theory iteratively from the data.	**Positive**<br>Self-Confidence<br>Feeling confident<br><br>Proactive during the consultation<br>Feel as though she can ask the therapist questions.<br><br>General Condition Management<br>Able to see improvements.<br><br>Feeling that "they matter"<br>Feeling that they matter, and that they are a real person.
6	Suman et al., 2017	Country<br>Netherlands<br><br>Participants<br>N = 44<br>59% Females / 41% Males<br>23 – 76 years old<br>LBP	(1)<br>Empathy, making the patient feeling cared for, understood, rapport<br>Videos from other LBP patients sharing their insights.	(4)<br>Explanation and Education<br>Aetiology of LBP.<br><br>Expected prognosis.<br><br>Tips and tricks on how to self-manage LBP.<br><br>12 short videos by surgeons, doctors, therapists.	This process evaluation was performed using the Linnan and Steckler framework and used a mixed method approach for data collection and analysis. The relationship between satisfaction of patients and exposure to the strategy was statistically examined. Semi-structured interviews were analysed using qualitative data analysis methods.	**Positive**<br>Patient satisfaction<br>Overall satisfaction<br><br>Enablement/Motivation<br>It is good to hear from someone that goes through this in his life, and who really has gotten better. It gives you more willpower to do it yourself as well.<br><br>Website alerted them the importance of exercise, which motivated them to start exercising.

						**Negative**<br>Not Feeling Understood<br>Some information on there was not applicable to their personal situation: things that they already knew, don't apply to them.

#	Source	Sample	Phenomenon of Interest		Design	Evaluation
			Affective Reassurance	Cognitive Reassurance		
7	Ree et al., 2014	Country<br>Denmark<br><br>Participants<br>10 Patients<br>30% Men / 70% Women<br>20-67 Years Old<br>Chronic Back Pain<br><br>Practitioner<br>Physiotherapists	(2)<br>Others<br>Comprehensible<br><br>Trusting the lecturers and perceiving them as experts	(2)<br>Explanations and Education<br>Using practical examples and images of the spine<br><br>Understanding why they felt the pain and that it was not sign of a serious disease	Focus Group<br><br>Systematic text condensation and descriptive cross-case analysis strategy.<br><br>Purposive sample	**Positive**<br>Self-Confidence, Enablement/Motivation, Acceptance of a Long Term Condition<br>Changing the participant's perception of how they could live with their complaints, exceeding their previous limits and dared to undertake activities they previously had avoided due to fear
8	Donovan and Blake, 2000	Country<br>UK<br><br>Participants<br>35 Patients<br>20% Men / 80% Women<br>New patients with suspected inflammatory arthropathy (RA, ankylosing spondylitis, etc.)<br><br>Practitioner<br>Consultant Rheumatologists	Not taking a patient's difficulties seriously when the diagnosis is uncertain or clear	(3)<br>Explanation and Education<br>Emphasizing the non-seriousness of osteoarthritis<br><br>Focusing on the early or mild nature of rheumatoid arthritis.<br><br>Explaining findings: wear and tear arthritis, creaking, that runs in families.	Semi-structured interviews, following a checklist of topics.<br><br>Grounded theory and ethnography.<br>Common themes identified and coded.<br><br>Theoretical sampling to compare conditions, until no new themes emerge.	**Negative**<br>Fear<br>Emphasis on the "early stage" of rheumatoid arthritis led to fears for the future, on patients who were already suffering enough, making them fear for the future.<br><br>Not feeling understood<br>Wanting doctor to be more concerned over health and welfare. This made patient's feel not understood on the seriousness of the symptoms they are living with.

#	Source	Sample	Phenomenon of Interest		Design	Evaluation
			Affective Reassurance	Cognitive Reassurance		
9	Hills and Kitchen, 2005	Country<br>UK<br><br>Participants<br>30 Patients<br>36-82 years old<br>30% Males / 70% Females<br>Acute-Chronic Musculoskeletal Condition (Fractures, Spondylosis, OA, Shoulder pain)<br><br>Practitioner<br>Physiotherapy	(4)<br>Making the patient feeling cared for<br>Praising the patient for their effort<br><br>Being seen quickly, regular appointments and opportunity to contact the department following discharge<br><br>Understood<br>A personal interest in the patient's well-being (being friendly and remembering things)<br><br>Empathy<br>Understanding, empathetic, knowledgeable	(5)<br>Education and Explanation<br>Modifying expectations of improvement at the beginning of treatment<br><br>Explanation of their problem and what improvement they are likely to make with treatment.<br><br>Explaining that their fracture is healed by the time they come in for treatment.<br><br>Devising home exercise regimens that incorporate functional activities.<br><br>Putting responsibility onto the patient	4 focus groups with purposeful sampling, with questions under the headings "opening, introductory/transition, key and ending questions", developed with reference to themes emerging from earlier developmental interviews in relation to pre-treatment, treatment and outcome of physiotherapy care.	**Positive**<br>Cognitive Reassurance: Enablement, Motivation, Self-Confidence<br>Explanation about their problem and what improvement they are likely to make. Increased confidence and motivation to persevere.<br><br>Self-Confidence<br>Reduced apprehension<br><br>Compliance<br>Improved compliance<br><br>Self-Efficacy<br>Awareness of the importance of self-efficacy<br><br>Satisfaction<br>Overall satisfaction

#	Source	Sample	Phenomenon of Interest		Design	Evaluation
			Affective Reassurance	Cognitive Reassurance		
10	Darlow et al., 2013	Country<br>New Zealand<br><br>Patients<br>12 Patients<br>Acute – Chronic Lower Back Pain<br>25% Males / 75% Females<br>18 – 55 years old<br><br>Practitioners<br>Wide range; including family doctors, physiotherapists, rheumatologists, osteopaths, acupuncturists, orthopaedic surgeon, massage therapist. Chinese medicine practitioner.	(1)<br>Attempt to Build Rapport<br>Casual manner – grinning, laughing, and using expletives towards the patient.	(5)<br>Explanation and Education<br>Simple diagnosis of a lumbar strain.<br><br>Explaining treatment around "aligning the back"<br><br>Educating patients on the importance of movement and activity positively<br><br>Educating on protecting the back with avoidance, posture and strengthening muscles<br><br>Pathoanatomic explanations	Interpretive Description: capturing themes and patterns within subjective perceptions.<br><br>Audio-recorded semi structured interviews.	**Negative**<br>Fear<br>Petrified about the seriousness of mis-alignment and damage to the spinal cord to paralyze her.<br><br>Fear<br>Patients interpreting the casual manner as odd, causing the patient to stop his physical work.<br><br>Fear, Frustration<br>Increased vigilance, worry, frustration, guilt and wanting to avoid movement.<br><br>**Positive**<br>Confidence<br>Increased confidence and less anxiety from the simple diagnosis<br><br>Belief Change: Importance of Exercise<br>Understanding the importance of moving

#	Source	Sample	Phenomenon of Interest		Design	Evaluation
			Affective Reassurance	Cognitive Reassurance		
11	Geraghty et al., 2019	<u>Country</u><br>UK<br><br><u>Participants</u><br>15 Patients<br>66.7% Females / 34.3% Males<br>18+, 59.8 years old mean age<br>LBP (acute, persistent, recurrent)	(6)<br><u>Making the Patient Feeling Cared For</u><br>Provided accountability<br><br><u>Understood</u><br>Supporting the realization that they can manage<br><br>Clear and easy to use information.<br><br><u>Making the patient feeling cared for</u><br>Email prompts to motivate patients to continue the exercise regimen.<br><br>Remote contact to a physiotherapist to clarify elements of online suggestions.	(4)<br><u>Education and Explanation</u><br>Education on pain, sleep, working with LBP, pain relief, daily living.<br><br>Specific use of activity for their LBP<br><br>Highlighting the centrality of self-management for LBP<br><br>Highlighting the importance of moving to ease the pain.<br><br><u>Relaxation</u><br>Remote contact from a physiotherapist that it's "all right" to keep moving.	Qualitative descriptive and interviews study with thematic analysis.	**Positive**<br><u>Self-Efficacy</u><br>Acknowledge the benefit of a multifaceted approach to self-management.<br><br><u>Self-Confidence</u><br>Deceased fear related to discomfort when doing activity for LBP<br><br><u>Behaviour Change: Physical Activity/ Exercise/ Movement</u><br>Increased planning to engage with, and maintain their physical activity, because the patient felt less afraid of activity<br><br>Behaviour changing form no movement to walking, stretching, moving when it hurts.<br><br><u>Motivation</u><br>Feeling supported and motivated by the Physiotherapist
12	Lærum et al., 2006	<u>Country</u><br>Norway<br><br><u>Participants</u><br>35 Patients<br>51% Males / 49% Females<br>23-65 Years Old	(1)<br><u>Understood</u><br>Adapting an explanation to concepts, knowledge and glossary by asking if the patient has had an explanation for their pain for example.	(6)<br><u>Explanation and Education</u><br>Positive feedback concerning strength and movement "your back seems to be strong… you move just fine"<br><br>Understandable explanation of why the patient is in pain	LBP consultations between a patient and practitioner , notes made on interaction . This was followed by 2 self-made summary scales, one called overall clinical competence (ranging from 0-10, 0= not good 10= very good), assessed on active	**Positive**<br><u>"Peace of Mind"</u><br>Patients getting an understandable explanation for the first time.<br><br><u>Being Taken Seriously</u><br>Feel like they're being taken more seriously

#	Source	Sample	Phenomenon of Interest		Design	Evaluation
			Affective Reassurance	Cognitive Reassurance		
		Chronic LBP, with Radiculopathy, 2 unverified serious spinal pathology<br><br><u>Doctors</u><br>11 males in neurology, rehabilitation medicine, orthopaedics, neurosurgery, and rheumatology.		Clear information that cancer or serious disease could be ruled out<br><br>Using simple explanations and metaphors “like having a leg cramp” or “wears and tears – what I call wrinkles. “prolapse will shrink like a grape shrinks to a raisin”.<br><br>Using plastic models of the spine, posters or scans when explaining pain.	listening nonverbally, verbally and para-verbally. Another scale then measured the patient’s thoughts, preferences, and feelings (patient-centredness).<br><br>After the consultation, a semi-structured interview was used, lasting 15-20 minutes, asking what the patient thought about the consultation, how satisfied (0-10), and the important characteristics of a “Good Back-Consultation”.	<u>Patient Satisfaction</u><br>Patient satisfaction = 7.8/10 in group A, which had higher patient centredness and overall clinical competence (8.1 and 7.4 respectfully), in comparison to Group B (6.6 and 7.2 respectfully).
13	Holt, Pincus and Vogel, 2015	<u>Country</u><br>UK<br><br><u>Participants</u><br>23 Patients<br>Aged 18 years old or older, 57 Years Old Average Age<br>56.5% Female / 43.5% Male<br>Acute – Chronic Non-specific low back pain<br><br><u>Practitioners</u><br>GPs	(5)<br><u>Understood</u><br>Taking the patient seriously (listening, understanding their story)<br><br>Feeling “known” by the doctor<br><br><u>Making the patient feeling cared for</u><br>Examination<br><br>Having the doctor doing everything they can do to help<br><br>Easy access to the GP and having time available for patients.	(5)<br><u>Explanations and Education</u><br>Ruling out serious disease<br><br>Explanations to what is contributing to the pain, physical explanations, advice on how to manage, prognosis, treatment explanation.<br><br>Having a vague estimation of prognosis<br><br>Having a choice in their treatment plan<br><br>Offering referrals	Interviewed at the GP surgery, using semi-structured interviews, focusing on what happened during consultations, and how their pain had since. Transcripts are analysed for the themes.	**Positive**<br><u>Feeling that they matter</u><br>Patients feel cared for and want to “get to the bottom of the situation” without pre-empting.<br><br><u>Trust for practitioner</u><br>Rapport or bond with the GP increased the patient’s trust for the GP.<br><br><u>Peace of mind</u><br>Patients felt peace of mind and grateful that it’s “nothing”<br><br><u>Self-efficacy</u><br>Greater sense of control when they understood why they had pain, prognosis, how to manage it.<br><br>**Negative**<br><u>Frustration</u><br>Vague explanations of prognosis led to frustration<br><br><u>Not feeling understood</u><br>Absence of reassuring behaviours

#	Source	Sample	Phenomenon of Interest		Design	Evaluation
			Affective Reassurance	Cognitive Reassurance		
14	Thomson and Collyer, 2017	Country<br>UK<br><br>Participants<br>9 patients<br>33% Females, 67% Males<br>Chronic LBP<br>Over 18 years old, 31-81 years old<br><br>Practitioners<br>Physiotherapists, Chiropractors, A/E, Osteopaths, GP, Rheumatologist, Surgeon	(2)<br>Empathy, Making the Patient Feeling Cared For<br>Caring for the person with trust, respect and empathy<br><br>Tutors walking in, talking to students as if the patient wasn't there)	(4)<br>Explanation and Education<br>Understanding of their pain<br><br>Using the word 'degeneration' of the disc<br><br>Analogies (donut with jam in between)<br><br>Jargonising	Interpretive qualitative research design, using constructivist grounded theory (coding, memo writing and diagramming) for data collection, analysis and conceptualisation, surrounding experiences and interpretations of explanations about their LBP, with semi-structured interviews.<br><br>Interviews are recorded, lasting 35-60 minutes, then transcribed verbatim.	**Positive**<br>Enablement/Motivation<br>Gaining an understanding of their LBP, having trust and confidence in explanations from the practitioner lead to higher engagement for decision making and personal behaviours for their own care.<br><br>Practitioner Trust<br>Alterations in beliefs and understanding of their pain made patients feel more confident in the practitioner's skills and knowledge<br><br>**Negative**<br>Feeling Old<br>Interpretation of the language used in explanations had a impact on fearful feelings "I'm turning into an old man, I'm not going to be able to do things I can do".<br><br>Degeneration was associated with dying like a tree, fading away – patients felt worried , fearful and no control over managing their LBP.<br><br>Less Engaged, Honest and Open<br>Jargonising resulted in some participants to lack the ability to understand or appreciate

						medical jargon, so they felt less engaged, less honest and open with the practitioner”.<br><br>Analogies (some) e.g., donut and jam, facilitated lower level of engagement “didn’t help me workout a way to fix it”<br><br>Tutor practitioners dismissive around feelings and concerns around medical words made the patient feel like they had a less active role in their care.

#	Source	Sample	Impact of Reassurance Mechanisms		Design	Impact of Reassurance
			Affective Reassurance	Cognitive Reassurance		
15	Moore et al., 2020	Country<br>UK<br><br>Participants<br>30 Patients<br>45 years old or older<br>50% Males and 50% Females<br>Knee pain and or stiffness in one or both knees with diagnosed OA.<br><br>Practitioners<br>Physiotherapists	(7)<br>Making the patient feeling cared for<br>Regular contact and support from family and friends<br><br>Convenient time and place for the exercise<br><br>Therapeutic alliance - collaboration, warmth and support between physiotherapists and participants. Including: mutual investment - equity in work (not letting the physio down), since the physiotherapist has put effort into caring for the patient.<br><br>Attentiveness to the other person.<br><br>Open, honest, trusted, listening.<br><br>Relaxation<br>Not rushing.<br><br>Motivation to keep going	(3)<br>Education about the benefits of exercise, how to perform, what should feel right and wrong<br><br>Advising patients to ask questions in return<br><br>Explanations offered and understood for self-efficacy with exercise	Longitudinal qualitative study with face-to-face-semi structured longitudinal interviews.<br><br>Layered thematic analysis on the views of exercises, physical activity, barriers and facilitators.	**Positive**<br>Motivation/Enablement, Behaviour Change: Physical Activity/Exercise/Movement<br>Affective reassurance facilitated exercise and physical activity in the long term<br><br>Fitting exercise into their daily lives "sitting at work, waiting for kettle to boil"<br><br>Reassurance linked to therapeutic alliance helped patients understand when pain was an indication to stop, and when it was safe to work through pain, which impacted on exercise and physical activity over 12 months later.

#	Source	Sample	Phenomenon of Interest		Design	Evaluation
			Affective Reassurance	Cognitive Reassurance		
16	Buus et al., 2014	Country<br>Denmark<br><br>Patients<br>25 Participants<br>56% Females / 44% Males<br>46.8 years old mean age<br>LBP<br><br>Practitioner<br>Counsellor	(2)<br>Understanding<br>Legitimizing the patient's experienced problems, by emphasizing problems were real, even though symptoms were not confirmed objectively by imaging.<br><br>Physical tests such as standing on toes.	(5)<br>Explanations and Education<br>Herniated disc: such as something toothpaste-like pressed against the discs that look like onion rings.<br><br>Exercise as a treatment<br><br>Changing work routines<br><br>Course of illness and sick role<br>There are many muscles we know little about and can't see, and that it's common to not have complete certainty about the cause of LBP.<br><br>Treatment<br>Recommended that it is healthy to do full body exercising several times a week, with cautions	Qualitative follow up interviews, including open-ended questions about "explanatory models of illness", experiences participating in the intervention and how it influenced everyday life and ability to work.<br><br>Interviews were digitally recorded and transcribed verbatim by research assistants.<br><br>Thematic analysis in stages (1) incident to incident, which had 14 distinct categories (2) open coding around 3 new categories in line with Kleinman's notion of explanatory models. (3) participant's perspective on receiving counselling (4) transcripts re-read, for balanced, nuanced interpretation of themes.	**Positive**<br>Enablement/Motivation<br>Some participants saw exercise as a way to strengthen a weak back<br><br>Enablement/Motivation<br>Encouraged to ask for changes at their work, with less strenuous work.<br><br>Condition Management<br>Emotionally calmed down<br><br>Self-Confidence<br>Self-confidence in own physical activities<br><br>Enablement/Motivation<br>Personal quest to improve physical scores<br><br>Belief Change: Importance of Exercise<br>Some would continue full body exercises until disability or complete obligations to make it impossible to do exercise<br><br>Self-Efficacy<br>Some would stop if they had no pain and continue if they had pain.<br><br>**Negative**<br>Found exercise as counterintuitive, as she [the

#	Source	Sample	Phenomenon of Interest		Design	Evaluation<br>patient] would lie passively on the couch to ease pain<br><br>Exercise could cause fragile
			Affective Reassurance	Cognitive Reassurance		
17	Barrett et al., 2018	Country<br>Ireland<br><br>Participants<br>23 Patients<br>65% Female, 45% Male<br>Over 18<br>Neck Pain<br><br>Practitioners<br>Outpatient Physiotherapists<br><br>Intervention<br>45 min classes of 4-6 participants – warmup, 10 exercises. Weekly. Plus: same exercise at home, every day.	(7)<br>Understood<br>Support, motivation and learning from peers.<br><br>Making the patient feeling cared for<br>Attentive nature of the physiotherapist, despite being a group.<br><br>The physiotherapist as an educator and facilitator<br><br>Therapeutic alliance	(2)<br>Education and Explanation<br>The physiotherapist as an educator and a facilitator<br><br>Systematic Belief Change<br>Changing beliefs about pain and exercise through education	Qualitative, semi-structured interviews	**Positive**<br>Reducing feeling of Isolation<br>Development of friendships. Increased determination and adherence with home exercise programming. Increased Motivation from a Shared Goal. Sharing common experiences and expertise.<br><br>Self-efficacy<br>Gained knowledge. Importance of pacing, controlling pain levels. Correcting for right technique. Increased self-management and responsibility.<br><br>Beliefs about pain and exercise<br>Exercise as a flare-management tool.<br><br>Embracing an active approach to care. Self-mediation. New beliefs. Slow process, may never resolve.<br><br>**Negative**<br>Under Pressure<br>Under pressure to keep pace with others. Uncomfortable due to being relatively younger.

#	Source	Sample	Phenomenon of Interest		Design	Evaluation
			Affective Reassurance	Cognitive Reassurance		
18	Mathias, Parry-Jones, and Huws, 2013	Country<br>UK<br><br>Participants<br>6 Patients<br>100% Females<br>Aged 46-64<br>Chronic, non-malignant pain<br><br>Intervention<br>PMP: acceptance-based pain management program: Cognitive Behavioural Therapy, Mindfulness and Acceptance Commitment Therapy, focused n pain-based education, pacing, goal setting and values, exercise/movement , mood management, mindfulness.	(2)<br>Empathy<br>I am not alone; others understand my pain.<br>Validation of their pain experience<br>Telling about other people's positive experiences<br><br>Understood<br>A new self – one with pain	(3)<br>I am not alone; others understand my pain.<br>Belief change regarding pain does not equal damage.<br><br>Psychoeducation of pain and mood cycles<br><br>Positive acceptance of pain education	Interpretative phenomenological Analysis	**Positive**<br>I am not alone.<br><br>Others Understand My Pain<br><br>Freedom from pain taking over.<br><br>A new self – one with pain<br>Self-confidence and Self-esteem<br><br>Exercise is possible<br><br>Mood and pain psychoeducation<br>Increased pain-related self-efficacy

#	Source	Sample	Phenomenon of Interest		Design	Evaluation
			Affective Reassurance	Cognitive Reassurance		
19	Olsen et al., 2016	Country<br>Norway<br><br>Participants<br>5 Patients<br>60% Males / 40% Females<br>52-78 years old<br>Chronic Hip Pain<br><br>Practitioner<br>Specialist Orthopaedic Physiotherapist following OA Management Clinical Guidelines	(2)<br>Creating Rapport<br>Inviting patients for questions, views, discussing topics regarding living with hip OA.<br><br>Understood<br>BBAT (Basic Body Awareness Therapy)<br>Reflective talk	(3)<br>Education<br>Hip Anatomy, Function, Hip OA Disease Processes.<br><br>Advice<br>Optimal Joint Loading Advice<br>Pain Interpretation<br>Physical Activity<br><br>Exercise Rx<br>Exercise demonstration with pictured pamphlets and exercise description	Qualitative, semi-structured interview with phenomenology	**Positive**<br>Becoming Motivated and Involved<br>From expert advice and peer support through dialogue<br><br>Movement Awareness Learning<br>From becoming aware of movement quality and experiencing own resources<br><br>Movement and Disease in a Long-Term Perspective<br>Movement awareness practice/integration and thoughts about the future

#	Source	Sample	Phenomenon of Interest		Design	Evaluation
			Affective Reassurance	Cognitive Reassurance		
20	Oosterhof et al., 2014	Country<br>Netherlands<br><br>Participants<br>16 Patients<br>Non-Specific or Specific Chronic Musculoskeletal Pain (more than 3 months)<br>75% Females and 25% Males<br>31 – 64 Years Old<br><br>Practitioners<br>N = 10 (Rehabilitation Physician, Social Worker, Psychologist, Physiotherapist, Occupational Therapist). Several years working with chronic pain patients, with additional pain rehab education.<br><br>Intervention<br>Multi-Disciplinary Rehabilitation Programme	(6)<br>Rapport<br>Taking the patient seriously<br><br>Practitioner is: tranquil, personal manner, being able to listen well.<br><br>Open Interaction with the patient and professional<br><br>Clear explanations<br><br>Organizational Problems<br>Cancelled appointments	(6)<br>**Shared Understanding Of pain**<br>Education<br>Explanation of Pain e.g., Pain does not = damage, psychosocial factors impacting pain.<br>Pain is related to muscles won't relax.<br><br>Learning New Behaviour<br>Talking about new behaviour by asking questions and prompting.<br><br>Maintaining New Behaviour<br>Advice regarding sport<br><br>Acknowledging for the physical part of the pain<br>Viewing and discussing test results	Qualitative research<br>Exploring: Patient's experience with pain and professionals and patient-professional interaction.<br><br>Interviews Post-Consultation, Therapy Sessions Observed.	**Positive**<br>Shared Understanding of the pain<br><br>Acknowledgement for the physical part of the pain<br><br>Learning New Behaviour<br><br>Maintaining New Behaviour<br><br>Being taken seriously<br><br>Being involved<br><br>Being Open<br><br>**Negative**<br>Organizational Problems<br><br>No Shared understanding<br>Resulting in patients being upset about how their pain is just all psychological.

#	Source	Sample	Phenomenon of Interest		Design	Evaluation
			Affective Reassurance	Cognitive Reassurance		
21	Matthias et al., 2012	Country<br>USA<br><br>Participants<br>26 Patients<br>Chronic MSK low back, cervical spine, hip, knee, shoulder interviewed.<br>Aged 24-62<br>15% Female / 85% Male<br><br>Intervention<br>12 contacts with nurse care manager<br>6: pain education, identifying pain influences, handling pain flare-ups, minimizing bed rest or inactivity, goal setting , relaxation, deep breathing.<br>Next 6: brief CBT.	(5)<br>Rapport<br>Shared goal setting<br>Listening<br>How do you feel?<br>Have you tried this?<br><br>Empathy<br>Understanding that there is a lot going on in patient's life, when having difficulty adhering to their treatment plan.	(4)<br>Helping patients find what works for their pain.<br>Reflecting by talking through pain triggers<br><br>Explanation<br>Encouragement - "Positivity: You're doing good, keep it up".<br>How to cope with pain<br><br>Education<br>Goal Setting	Grounded Theory. Face-to-face interviews, audio recorded.	**Positive**<br>Shared Goal Setting =<br>Feeling Accountable, appear "Smart" and "Well-Prepared" when talking to the nurse<br><br>How do you feel?<br>Can talk to somebody about it – cope with the pain better.<br><br>Have you tried this?<br>They're there to talk about your pain – like a pain management counsellor.<br><br>Listening = feeling cared for, stress relief, knowing someone was trying to make her feel better<br><br>Empathy<br>Not feeling judged<br><br>Explanation<br>Encouragement = motivation, feeling like an "all-star"<br><br>How to go about coping with Pain = reduced frustration, having someone helped

#	Source	Sample	Phenomenon of Interest	Design	Evaluation

#	Source	Sample	Phenomenon of Interest		Design	Evaluation
			Affective Reassurance	Cognitive Reassurance		
22	Andersen et al., 2014<br><br>"Here we're all in the same boat"--a qualitative study of group-based rehabilitation for sick-listed citizens with chronic pain<br><br>https://pubmed.ncbi.nlm.nih.gov/24730653/	<u>Country</u><br>Denmark<br><br><u>Participants</u><br>7 Patients<br>Age 33 – 57 years old<br>43% Females / 57% Males<br>Musculoskeletal Pain<br><br><u>Practitioners</u><br>Physiotherapists	(2)<br><u>Rapport</u><br>Highly participative/mutually supportive<br><br>Group Discussions – highly empathetic and open	(8)<br><u>Explanation/ Education</u><br>(1)Techniques to deal with frustration, fatigue, isolation, poor sleep. (2) Appropriate exercise. (3) Appropriate Medication Use (4) Effective Communication with (5) Optimal Nutrition (6) Pacing activity and rest (7) Evaluating New Treatment (8) Self-Efficacy Course	Phenomenological-Hermeneutical Approach; systematic text condensation, descriptive and explorative, for thematic cross-case analysis.<br><br>7 semi-structured interviews, face-to-face in an undisturbed room. Translated from Danish to English by a "professional translator". Reflexive Listening used.<br><br>Maximum variation sampling to capture diversity on pain localization, sex, age, degree of participation.	**<u>Positive</u>**<br>**Affective**<br><u>Rapport</u><br>Group Discussions = all the people in the group were in the same situation, living with pain, advice from other participants on where to get assistive devices.<br>Group Training = enhanced motivation<br><br><u>Empathy</u><br>Instructors were not "Arnold Schwarzenegger's, without pain anywhere"<br><br>**Cognitive**<br>New ability to overcome periods of difficulty through awareness of the psychological factors involved.<br><br>Goal setting = Doing things that they want to do, rather that they "ought" to do, overcoming feelings of guilt and guilty conscience.<br><br>**<u>Negative</u>**<br>**Affective**<br><u>Empathy</u><br>Listening to others about their pain made him more unwell, because he felt they were more unwell than he was.<br><br><u>Explanation/Education</u><br>Frustrated, as; "wouldn't have the professional input to get the kind of things across to me that "I" need".<br>No use: "because I know everything".
#	Source	Sample	Phenomenon of Interest		Design	Evaluation

			Affective Reassurance	Cognitive Reassurance		
23	Cederbom, Nortvedt and Lilekroken, 2020	Country<br>Norway<br><br>Participants<br>12 Patients<br>79-96 years old<br>75% Females /<br>25% Males<br>Chronic MSK pain	(5)<br>Rapport<br>Easy to talk to.<br><br>Promoting good teamwork between participants and the PT.<br><br>Continuous shared decision-making.<br><br>Empathy<br>Exercise that the patient finds fun.<br>Caring towards the participant's needs	(4)<br>Explanation<br>Behavioural change maintenance strategies<br><br>Education<br>Individualized, fun exercises.<br>Expert knowledge.	Inductive, explorative qualitative design<br><br>Semi-structured interviews.	**Positive**<br>**Affective**<br>Easy to talk to, co-operate and discuss social situations with.<br><br>**Cognitive**<br>Feeling understood by a healthcare professional.

Table S3: Quality assessment of included studies using CASP Qualitative Checklist

Study	Clear statement of aims?	Qualitative methods appropriate?	Research design appropriate to address aims?	Recruitment strategy appropriate to aims?	Data collected in a way that addressed research issue?	Relationship between researcher and participants adequately considered?	Ethical issues taken into consideration?	Data analysis sufficiently rigorous?	Clear statement of findings?	Does the research have practical application?	Does it add to our understanding?
(Dures et al., 2016)											
(Vinall-Collier, Madill, and Firth, 2017)											
(Haugli, Strand and Finset, 2004)											
(Saunders et al., 2020)											
(Kidd, Bond, and Bell, 2011)											
(Suman et al., 2017)											
(Ree et al., 2014)											
(Donovan and Blake, 2000)											
(Shaw et al., 2011)											
(Hills and Kitchen, 2007)											
(Darlow et al., 2013)											

(Geraghty et al., 2019)											
(Lærum et al., 2006)											
(Holt, Pincus and Vogel, 2015)											
(Thompson and Katie, 2017)											
(Moore et al., 2020)											
(Buus et al., 2014)											
(Barrett et al., 2018)											
(Miller, Litva and Gabbay, 2009)											
(Mathias, Parry-Jones, and Huws, 2013)											
(Olsen et al., 2016)											
(Oosterhof et al., 2014)											

Table S3 expanded: Quality assessment of included studies using MMAT Criteria

Study	Are there clear research questions?	Do the collected data allow to address the research questions?	Is there an adequate rationale for using a mixed methods design to address the research question?	Are there different components of the study effectively integrated to answer the research question?	Are the outputs of the integration of qualitative and quantitative components adequately interpreted?	Are divergences and inconsistencies between quantitative and qualitative results adequately addressed?	Do the different components of the study adhere to the quality criteria of each tradition of the methods involved?
(Lærum et al., 2006)							
(Vinall-Collier, Madill, and Firth, 2016)							

Legend

	Yes
	Can't Tell
	No

Table S4: The impact of reassurance styles

CERQual Summary of Thematic Findings – Positive Impact Affective Reassurance Mechanisms

#	Review Findings		Studies Contributing to Review Findings	CERQual assessment of confidence in evidence	Explanation of CERQual assessment
	Themes	Subthemes/Codes			
1	Therapeutic Relationship Building and Interpersonal Skills	Interpersonal skills, relationship building: mutual support, activating and partnering, open, honest trusted listening, shared decision making	1. Vinall-Collier, Madill and Firth, 2016<br>2. Haugli, Strand and Finset, 2004<br>3. Hills and Kitchen, 2005<br>4. Thomson and Katie, 2017<br>5. Moore et al., 2020<br>6. Barrett et al., 2018<br>7. Oosterhof et al., 2014<br>8. Andersen et al., 2014<br>9. Cederbom, Nortvedt, and Lilekroken, 2020	High	Fourteen studies contribute to this finding. No or very minor methodological and relevance concerns. Moderate concerns for adequacy due to thin data. No or very minor concerns for coherence.
		Stories from other MSK patients sharing their insights and experiences	1. Suman et al., 2017<br>2. Barrett et al., 2018<br>3. Andersen et al., 2014		
		Encouragement, transparency of progress and outcome, encouragement	1. Kidd, Bond, and Bell, 2011<br>2. Geraghty et al., 2019		
		Taking the patient seriously (Listening, understanding their story)	1. Holt, Pincus and Vogel, 2015<br>2. Oosterhof et al., 2014		
		Supporting regular contact with family and friends	1. Moore et al., 2020<br>2. Barrett et al., 2018		
		Supporting regular contact with family and friends	1. Moore et al., 2020<br>2. Barrett et al., 2018		
		Expert educators and practitioners used clear comprehensible language	1. Ree et al., 2014<br>2. Oosterhof et al., 2014		
		Praising patient for their effort	1. Hills and Kitchen, 2005<br>2. Matthias et al., 2012		
		Practitioner providing accountability with email prompts	1. Geraghty et al., 2019		

		Neither not too positive or negative	1. Haugli, Strand and Finset, 2004		
		Understanding there is a lot going on in the patient's life, causing problems with exercise adherence	1. Matthias et al., 2012		
2	Individualized Patient-Centred Approach	Appreciating the holistic impact of the condition	1. Dures et al., 2016<br>2. Lærum et al. 2006	Moderate	Five studies contribute to this finding. Moderate methodological concerns from inadequate relationship between researcher and participant relationship. Minor concerns regarding coherence and relevance concerns. Moderate concerns for adequacy, as there is thin data for each code.
		Individual not disease entity	1. Haugli, Strand and Finset, 2004		
		Adapting explanations to patient's existing understanding of their condition	2. Lærum et al. 2006		
		Believing what the patient was experiencing is real	1. Buus et al., 2014<br>2. Barrett et al., 2018		
3	Appointment Organization	Routine consultations	1. Dures et al., 2016<br>2. Hills and Kitchen, 2005	Low	Six studies contribute to this finding. Minor methodological limitation concerns due to inadequate consideration of the researcher-participant relationship in one study. No or very minor concerns for relevance and coherence of data. Moderate concerns for adequacy of data due to thin data.
		Able to contact practitioner when symptoms exacerbate	1. Haugli, Strand and Finset, 2004		
		Enough dialogue time for care	1. Haugli, Strand and Finset, 2004		
		Short MRI waiting times	1. Saunders et al., 2020		
		Opportunity to contact following discharge, seen quickly	1. Hills and Kitchen, 2005		
		Remote contact with Physiotherapist to clarify elements of online suggestions	1. Geraghty et al., 2019		
		Easy access to GP and time available for patients	1. Holt, Pincus and Vogel, 2015		
4	Practical Skills	Examination	1. Holt, Pincus and Vogel, 2015	Very Low	Three studies contribute to this finding. Moderate methodological limitations from research-participant relationship. No or very minor concerns for
		Not rushing	1. Moore et al., 2020		

		Physical exam	1. Buus et al., 2014		coherence and relevance. Serious concerns for adequacy of data.

Table S5. CERQual Summary of qualitative findings – Positive Impact Cognitive Reassurance Mechanisms

#	Review Findings		Studies Contributing to Review Findings	CERQual assessment of confidence in evidence	Explanation of CERQual assessment
	Themes	Subthemes/Codes			
1	Disease Education	Information about disease and prognosis, diagnosis/testing, aetiology, exercise, treatment	1. Andersen et al., 2014<br>2. Barrett et al., 2018;<br>3. Buus et al, 2014;<br>4. Haugli, Strand and Finset, 2004;<br>5. Hills and Kitchen, 2005;<br>6. Lærum et al., 2006;<br>7. Holt, Pincus and Vogel, 2015;<br>8. Matthias et al., 2012;<br>9. Oosterhof et al., 2014;<br>10. Suman et al., 2017;<br>11. Thomson and Katie, 2017;	High	Ten studies contribute to this finding. No or very minor concerns regarding adequacy of data. Moderate concerns regarding coherence, due to the variety of education modalities. No or very minor concerns for relevance. Minor concerns for methodological limitations due to inadequate consideration of the patient-practitioner relationship.
		Ruling out sinister pathology	1. Lærum et al., 2006;<br>2. Holt, Pincus and Vogel, 2015		
		Positivity regarding pain (e.g. pain does not always equal damage)	1. Kidd, Bond, and Bell, 2011;<br>2. Oosterhof et al., 2014;<br>3. Barrett et al., 2018		
		Asking patients to ask questions in return	1. Moore et al., 2020;<br>2. Olsen et al., 2016;<br>3. Oosterhof et al., 2014		
		Practical examples, models images of spine	1. Ree et al., 2014;<br>2. Lærum et al., 2006		
		Importance of moving	1. Darlow et al., 2013;<br>1. Geraghty et al., 2019		
		Movement Feedback "strong, moving fine"	1. Lærum et al., 2006		
		Metaphors and explanations "like leg cramp" "wears and tears are like wrinkles" "prolapse will shrink like a raisin"	1. Lærum et al., 2006		
		"We're going to do everything we can to help you and get to the bottom of this"	1. Holt, Pincus and Vogel, 2015		
		Referrals	1. Holt, Pincus and Vogel, 2015		

		Simple diagnosis "lumbar strain"	1. Darlow et al., 2013		
		Psychoeducation of pain and mood cycles	1. Barrett et al., 2018		
		Behavioural change strategies	1. Cederbom, Nortvedt, and Lilekroken, 2020		
2	Self-Management	Convenient and fun self-help exercises involving functional activities and how to do them	1. Andersen et al., 2014;<br>2. Buus et al., 2014;<br>3. Cederbom, Nortvedt, and Lilekroken, 2020<br>4. Hills and Kitchen, 2005;<br>5. Kidd, Bond, and Bell, 2011;<br>6. Matthias et al., 2012;<br>7. Moore et al., 2020;	High	Ten studies contribute to this finding. No or very minor concerns regarding adequacy of data. Moderate concerns regarding coherence of data, as self-management combinations are relatively ambiguous. No or very minor concerns regarding relevance of data. Minor concerns regarding methodological limitations.
		Self-Management Combinations (Exercise, Pain Relief)	1. Dures et al., 2016;<br>2. Holt, Pincus and Vogel, 2015;<br>3. Barrett et al., 2018;<br>4. Suman et al., 2017;<br>5. Vinall-Collier, Madill, and Firth, 2016		
		Pamphlets and pictures with exercise description	1. Barrett et al., 2018;<br>2. Matthias et al., 2012		

3	Expectation Modification with goal setting and education	1. Hills and Kitchen, 2005;<br>2. Matthias et al., 2012	Low	Two studies contribute to this finding. Serious concerns for adequacy of data. No or very minor concerns for coherence and relevance.

Table S6. CERQual Summary of qualitative findings – Negative Impact Affective Reassurance Mechanisms

#	Review Finding		Studies Contributing to Review Findings	CERQual assessment of confidence in evidence	Explanation of CERQual assessment
	Themes	Subthemes/Codes			
1	Casual Manner	Grinning, laughing using expletives to patient	1. Darlow et al., 2013	Low	Three studies contribute to this finding. No or very minor concerns for methodological limitations and relevance. Serious concerns for adequacy of data. Minor concerns for coherence as laughing with the patient contradicts partnership in “therapeutic relationship building”
		Supervisors walking in to talk to tutees and not acknowledging the patient	2. Thomson and Katie, 2017		

#	Themes	Subthemes/Codes	Studies Contributing to Review Findings	CERQual assessment of confidence in evidence	Explanation of CERQual assessment
		Not taking a patient and their difficulties seriously when there is a unclear or un-specific diagnosis	3. Donovan and Blake, 2000		
2	Empathy<br>Listening to others about their pain in a group low back pain talking therapy session		1. Andersen et al., 2014	Very Low	One study contributes to this finding. No or very minor concerns for methodological limitations and relevance. Minor concerns for coherence, as listening to other patient's success stories and journey is a positive impact code
3	Organization<br>Appointment cancellation		1. Oosterhof et al., 2014	Very Low	One study contributes to this finding. No or very minor concerns for methodological limitations, relevance and coherence. Serious concerns for the adequacy of data.
4	Group sessions<br>Under pressure to keep pace with other individuals in a group exercise session due to others being "younger"		1. Barrett et al., 2018	Very Low	One study contributes to this finding. No or very minor concerns for methodological limitations, relevance and coherence. Serious concerns for the adequacy of data.

Table S7. CERQual Summary of qualitative findings – Negative Impact Cognitive Reassurance Mechanisms

#	Review Findings		Studies Contributing to Review Findings	CERQual assessment of confidence in evidence	Explanation of CERQual assessment
	Themes	Subthemes/Codes			
1	Pathoanatomic and avoidance prognostic education	Emphasizing the non-seriousness of osteoarthritis and not acknowledging the suffering the individual is living with	1. Donovan and Blake, 2000	Moderate	Seven studies contribute to this finding. Minor concerns for methodological limitations due to relationship between researcher and participants not adequately considered. Moderate concerns for adequacy of data due to thin data for each code. No or very minor concerns for coherence and relevance of the data.
		Focusing on the early or mild nature or rheumatoid arthritis despite the patient already suffering a lot	1. Donovan and Blake, 2000		
		Vague estimation of the prognosis	1. Holt, Pincus and Vogel, 2015		

		Protecting the back with avoidance and pathoanatomic explanations	1. Darlow et al., 2013		
		Importance of alignment therapy	1. Darlow et al., 2013		
		Degeneration of the intervertebral discs	1. Thomson and Katie, 2017		
		Pathoanatomic analogies e.g. donut and jam for the intervertebral discs	1. Thomson and Katie, 2017		
2	Education/Advice to exercise as a treatment rather than resting	Exercise is counterintuitive as rest would ease the patient's pain	1. Buus et al., 2014	Very Low	One study contributes to this finding. Moderate concerns for methodological limitations due to the relationship between researcher and participants not adequately considered. Serious concerns for adequacy of data. No or very minor concerns for coherence and relevance.
3	Psychosocial factors education	Explaining the psychosocial contributors to pain	1. Oosterhof et al., 2014	Very Low	One study contributes to this finding. Very minor or no concerns for methodological limitations, coherence and relevance. Serious concerns for the adequacy of data.

Impact of Reassurance

Table S8. CERQual Summary of Thematic findings – Positive Impact of Reassurance

#	Review Findings		Studies Contributing to review Findings	CERQual assessment of confidence in evidence	Explanation of CERQual assessment
	Themes	Subthemes/Codes			
1	Patient Confidence	Proactive during the consultation	1. Dures et al., 2016<br>2. Kidd Bond and Bell, 2011	High confidence	Twenty-two studies contribute to this finding. Minor concerns regarding methodological limitations, coherence, and relevance. However, minor

		Patient participation	1. Vinall-Collier, Madill, and Firth, 2016<br>2. Oosterhof et al., 2014<br>3. Andersen et al., 2014		concerns regarding adequacy, as subthemes, “feeling that they matter” and “being taken seriously” has thin data.
		Enablement/Motivation	1. Suman et al., 2017<br>2. Ree et al., 2014<br>3. Hills and Kitchen, 2005<br>4. Geraghty et al., 2019<br>5. Holt, Pincus and Vogel, 2015<br>6. Moore et al., 2020<br>7. Buus et al., 2014<br>8. Barrett et al., 2018<br>9. Olsen et al., 2016<br>10. Matthias et al., 2012		
		Self-confidence	1. Kidd Bond and Bell, 2011<br>2. Ree et al., 2014<br>3. Darlow et al., 2013<br>4. Buus et al., 2014<br>5. Mathias, Parry-Jones and Huws, 2013		
		Feeling of security	1. Haugli, Strand and Finset, 2004<br>2. Saunders et al., 2020		
		Feeling that “they matter”	1. Kidd Bond and Bell, 2011		
		Being taken seriously	1. Lærum et al., 2006<br>2. Oosterhof et al., 2014		
2	Condition Management	Seeing Improvements	1. Dures et al., 2016<br>2. Barrett et al., 2018	High confidence	Twenty-one studies contribute to this finding. Minor concerns regarding methodological limitations. No or very minor

		Feeling Understood	1. Vinall-Collier, Madill, and Firth, 2016<br>2. Buus et al., 2014<br>3. Matthias et al., 2012<br>4. Cederbom, Nortvedt and Lilekroken, 2020<br>5. Lærum et al., 2006<br>6. Thomson and Katie, 2017<br>7. Oosterhof et al., 2014		concerns regarding coherence, and relevance. Moderate concerns regarding adequacy due to four single study findings.
		Pain reduction	1. Haugli, Strand and Finset, 2004		
		Belief Change: Importance of exercise	1. Darlow et al., 2013<br>2. Buus et al., 2014<br>3. Barrett et al., 2018<br>4. Mathias, Parry-Jones and Huws, 2013		
		Behaviour Change: Physical Activity/Exercise/Movement	1. Geraghty et al., 2019<br>2. Thomson and Katie, 2017<br>3. Moore et al., 2020<br>4. Oosterhof et al., 2014		
		"Peace of Mind"	1. Holt, Pincus and Vogel, 2015<br>2. Matthias et al., 2012		
		Self-efficacy	1. Thomson and Katie, 2017<br>2. Moore et al., 2020<br>3. Holt, Pincus and Vogel, 2015<br>4. Mathias, Parry-Jones and Huws, 2013<br>5. Matthias et al., 2012<br>6. Andersen et al., 2014<br>7. Hills and Kitchen, 2005		

3	Acceptance of a Long-Term Condition	Acceptance	1. Dures et al., 2016<br>2. Ree et al., 2014<br>3. Olsen et al., 2016	Moderate Confidence	Three studies contribute to this finding. No or very minor concerns regarding methodological limitations. Minor concerns regarding adequacy. No/very minor concerns for coherence and relevance.
		Feeling Accountable	1. Matthias et al., 2012		
		Reduced frustration	1. Matthias et al., 2012		
4	Patient Satisfaction		1. Suman et al., 2017<br>2. Hills and Kitchen, 2005<br>3. Lærum et al., 2006	Moderate Confidence	Three studies contribute to this finding. Minor concerns regarding methodological limitations. Minor concerns regarding coherence, adequacy, and relevance.
5	Trust	Practitioner	1. Holt, Pincus and Vogel, 2015<br>2. Thomson and Katie, 2017	Moderate Confidence	Four studies contributing to this finding. No or very minor concerns regarding methodological limitations, coherence and relevance. Moderate concerns regarding adequacy of data, due to thin data.
		Not feeling judged	1. Matthias et al., 2012		
		More Open and Honest	1. Cederbom, Nortvedt and Lilekroken, 2020		
6	Reduced Feeling of Isolation		1. Barrett et al., 2018<br>2. Matthias, Parry-Jones and Huws, 2013<br>3. Andersen et al., 2014	Moderate Confidence	Three studies contribute to this finding. No or very minor concerns regarding methodological considerations, coherence, and relevance. Minor concerns regarding adequacy.
7	Compliance		1. Hills and Kitchen, 2005	Very Low Confidence	One study contributes to this finding. No or very minor concerns regarding methodological limitations, coherence and relevance. Serious concerns regarding adequacy.

Table S9 CERQual Summary of Thematic Findings – Negative Impact of Reassurance

#	Review Findings		Studies Contributing to Review Findings	CERQual assessment of confidence in evidence	Explanation of CERQual assessment
	Themes	Subthemes/Codes			
1	Fear		1. Donovan and Blake, 2000<br>2. Darlow et al., 2013<br>3. Thomson and Katie, 2017	Moderate confidence	Three studies contribute to this finding. No or very minor concerns regarding methodological limitations, relevance and coherence. Moderate concerns regarding adequacy due to limited data.
2	Poor Condition Management	Exercise perceived as counterintuitive	1. Buus et al., 2014	Low confidence	Two studies contribute to this finding. No or very minor concerns regarding methodological limitations. Minor concerns regarding coherence and relevance. Moderate concerns regarding adequacy due to limited studies.
		Feeling unwell	1. Andersen et al., 2014		
3	Not feeling understood		1. Donovan and Blake, 2000<br>2. Oosterhof et al., 2014	Low confidence	Two studies contribute to this finding. Minor concerns regarding methodological limitations, based on researcher's relationship with participants. No or very minor concerns regarding, coherence, and relevance. Moderate concerns regarding adequacy due to limited data.
4	Frustration		1. Holt, Pincus and Vogel, 2015<br>2. Andersen et al., 2014	Low confidence	Two studies contribute to this finding. No or very minor concerns regarding, methodological limitations, coherence, and relevance. Moderate concerns regarding adequacy due to limited data.
5	Feeling Old		1. Thomson and Katie, 2017	Very Low confidence	One study contributes to this finding. No or very minor concerns regarding methodological limitations, coherence, and relevance. Moderate concerns regarding adequacy due to a single study.
6	Less Engaged, Honest and Open		1. Thomson and Katie, 2017	Very Low confidence	One study contributes to this finding. No or very minor concerns regarding methodological limitations, coherence, and relevance. Moderate concerns regarding adequacy due to a single study.
7	Under Pressure		1. Barrett et al., 2018	Very Low confidence	One study contributes to this finding. No or very minor concerns regarding methodological limitations. Moderate concerns regarding relevance, coherence, and adequacy.

Legend: CERQual Confidence Colour Codes

Very Low
Low
Moderate
High

Paragraph S1: Additional themes, subthemes/codes with thin data

(1) For Patient Confidence Theme: *Feeling that "they matter"*, which was contributed by the physiotherapist's ability to communicate their understanding of the patient's condition, as well as education and self-help advice (Kidd, Bond and Bell, 2011). (2) *"Being taken seriously"*, was related to having explanations and concepts adapted towards patients, providing clear information and education regarding the pain's aetiology, as well as listening to the patient carefully (Oosterhof et al, 2014; Lærum, 2006). (3) *Being proactive during the consultation* increased as a result of practitioner's ability to use patient-centred communication techniques, rather than didactic approaches (Dures et al., 2016; Kidd, Bond, and Bell, 2011). (4) *Feeling of security: (explain this)* supported by 2 studies (Haugli, Strand and Finset, 2004; Saunders et al, 2020).

(2) For Condition Management Theme: (1) *Peace of mind with stress relief,* when patients learn that they do not have a sinister disease, and when they are listened to (Holt, Pincus and Vogel, 2015; Matthias et al., 2012). (2) *Feeling accountable and less frustrated*, after patients have a shared-goal setting and pain coping discussion with their practitioner (Matthias et al., 2012). (3) *Increased general confidence in managing their condition and associated pain*, when practitioners highlighted the value and believed in the patient's expertise of the patient's condition and management experience (Dures et al., 2016; Barrett et al., 2018; Haugli, Strand and Finset, 2004).

(3) For Trust Theme: Patients felt that their practitioner was more trustworthy after building greater rapport or bond with their practitioner, and after greater understanding of their condition after the practitioner's explanations (Holt, Pincus and Vogel, 2016; Thomson and Katie, 2017). As a result, greater trust towards practitioners lead to higher engagement during decision making and self-efficacy (Thomson and Katie, 2017). Moreover, *not feeling judged* was associated with practitioners empathizing with the patient, which also contributed to building rapport with the patient (Matthias et al., 2012).

(4) For Reduced Feeling of Isolation Theme: Furthermore, with patients "all in the same boat", rapport across patients was strong (Andersen et al., 2014). This also increased the determination to adhere to home exercise programming from a shared goal with their peers (Barrett et al., 2018).

(5) For Compliance Theme: On the other hand, in an individual setting, Hills and Kitchen (2005) found that compliance to a home exercise program improved by affective reassurance including: patient praising, friendliness, empathy, knowledge and regular appointments. Furthermore, cognitive reassurance including: modifying expectations, explanations and putting the responsibility on the patient. However, some styles of reassurance contradicted these seven themes of positive findings.

(6) Poor Condition Management: Two subthemes were identified within poor condition management: *exercise perceived as counterintuitive and feeling unwell*. The first subtheme was due to patients not understanding the role of exercise and the meaning of pain. This is as patients found rest to ease the pain, making it an appropriate intervention, whereas exercise increased the pain, therefore making it less intuitive (Buus et al., 2014). Secondly, Andersen et al. (2014) found that one patient felt more unwell after listening to others in worse pain.

(7) Not feeling understood: Patients didn't feel understood after consultant rheumatologists did not take a patient's difficulties and condition seriously, even though the patients felt that their condition was quite serious (Donovan and Blake, 2000). Additionally, some patients felt a reduced perception of a shared understanding of the patient's condition, due to cognitive reassurance of explaining the large psychosocial contribution towards the patient's pain (Oosterhof et al., 2014).

(8) Frustration: Overall, patients perceived cognitive reassurance to cause frustration. Patients found that vague explanations and estimations of their prognosis to recover or improve from their pain led to frustration upon consultation exit (Holt, Pincus and Vogel, 2015). Differently, Andersen et al. (2014) found that some patients became frustrated because professional input was 'no use' to him, due to not learning anything new from explanations and education regarding personalized condition information and self-management.

(9) Theme 5: Feeling Old and Theme 6: Less Engaged, Honest and Open: Both of these themes were only supported by one article: Thomson and Katie (2017). Patients' interpretation of specific language used by healthcare professionals made them feel old and anxious about the future. This included: degeneration, which patients associated with dying like a tree, fading away and not being able to do things they are currently able to do. Furthermore, using medical jargon with patients resulted in some patients not understanding their pain, which made them less engaged and unwilling to be open to the practitioner about their complaint (Thomson and Katie, 2017).

(10) Under Pressure: Lastly, when in a group setting with individuals at a better health condition baseline, some patients felt under pressure to "keep pace" with the rest of the group (Barrett et al., 2018). Also, negative beliefs around their condition, such

as “exercise could cause fragile joints to disjoint”, also placed pressure on patients due to fear of worsening their presenting complaint (Buus et al., 2014).
